# Transcriptome Profiling of Light-Regulated Anthocyanin Biosynthesis in the Pericarp of Litchi

**DOI:** 10.3389/fpls.2016.00963

**Published:** 2016-06-29

**Authors:** Hong-Na Zhang, Wei-Cai Li, Hui-Cong Wang, Sheng-You Shi, Bo Shu, Li-Qin Liu, Yong-Zan Wei, Jiang-Hui Xie

**Affiliations:** ^1^Key Laboratory of Tropical Fruit Biology, Ministry of Agriculture, South Subtropical Crops Research Institute, Chinese Academy of Tropical Agricultural SciencesZhanjiang, China; ^2^College of Horticulture, South China Agricultural UniversityGuangzhou, China

**Keywords:** Litchi (*Litchi chinensis* Sonn.), transcriptome, light, photoreceptors, anthocyanin biosynthesis

## Abstract

Light is a key environmental factor that affects anthocyanin biosynthesis. To enhance our understanding of the mechanisms involved in light-regulated anthocyanin biosynthesis in the pericarp of litchi, we performed transcriptomic analyses on the basis of Illumina sequencing. Fruit clusters were bagged with double-layer Kraft paper bags at 42 days after anthesis. The bags were removed after 2 weeks. Under light conditions, anthocyanins accumulated rapidly in the pericarp. RNA sequences were *de novo* assembled into 75,935 unigenes with an average length of 913 bp. Approximately 74.5% of unigenes (56,601) were annotated against four public protein databases. A total of 16,622 unigenes that significantly differed in terms of abundance were identified. These unigenes are implicated in light signal perception and transduction, flavonoid biosynthesis, carotenoid biosynthesis, plant hormone signal transduction, and photosynthesis. In photoreceptors, the expression levels of UV RESISTANCE LOCUS 8 (UVR8), Phototropin 2 (PHOT2), Phytochrome B (PHYB), and Phytochrome C (PHYC) increased significantly when the fruits were exposed to light. This result indicated that they likely play important roles in anthocyanin biosynthesis regulation. After analyzed digital gene expression (DGE), we found that the light signal transduction elements of COP1 and COP10 might be responsible for anthocyanin biosynthesis regulation. After the bags were removed, nearly all structural and regulatory genes, such as UDP-glucose: flavonoid-3-*O*-glucosyltransferase (UFGT), MYB, basic helix-loop-helix (bHLH), and WD40, involved in the anthocyanin biosynthetic pathway were upregulated. In addition to MYB-bHLH-WD40 transcription complex, ELONGATED HYPOCOTYL (HY5), NAM/ATAF/CUC (NAC), homeodomain leucine zipper proteins (ATHBs), and FAR-RED ELONGATED HYPOCOTYL (FHY) possibly participate in light-induced responses. On the basis of DGEs and qRT-PCR validation, we observed a light-induced anthocyanin biosynthesis and regulation pattern in litchi pericarp. This study enhanced our understanding of the molecular mechanisms governing light-induced anthocyanin biosynthesis in litchi pericarp.

## Introduction

Litchi (*Litchi chinensis* Sonn.), a member of the Sapindaceae, is an important subtropical fruit crop, which is indigenous to South China. Litchi fruit displays a typical red appearance attributed to anthocyanin accumulation and chlorophyll degradation in its pericarp (Lai et al., 2015). The structural gene *LcUFGT* and the transcription factor (TF) *LcMYB1* play major roles in anthocyanin biosynthesis (Wei et al., 2011; Lai et al., 2014; Li et al., 2015). However, anthocyanin biosynthesis is complex pathway, which regulated by a suite of TFs and modulated by environmental factors. Light is one of the most important environmental factors regulating anthocyanin biosynthesis. However, the actual signal transduction pathways of light-enhanced anthocyanin accumulation in litchi are not yet well defined.

Fruit color is a considerable exterior quality, which is mainly attributed to anthocyanins, chlorophylls, and carotenoids (Macheix et al., 1990). Anthocyanins, which are synthesized via the flavonoid pathway, are the main pigments that determine fruit coloration in litchi (Wei et al., 2011). Light exposure increases, while shading decreases the concentration of anthocyanins in fruits (Takos et al., 2006; Wei et al., 2011; Azuma et al., 2012). Light-regulated anthocyanin biosynthesis and distribution are associated with light perception and signal transduction (Jaakola, 2013).

Under light conditions, specific plant photoreceptors receive light signal and then form a cascade of intracellular second messenger systems by transducing signals to regulate anthocyanin synthesis (Jaakola, 2013). Light signals ranging from UV-A to far red are perceived by three kinds of classical photoreceptors, such as phytochromes (PHYs), cryptochromes (CRYs), and phototropins (PHOTs) (Li et al., 2012). UV-B-specific UVR8 is a key regulator of UV-B responses, especially photomorphogenesis and flavonoid biosynthesis induction (Rizzini et al., 2011; Christie et al., 2012). Once activated by light, photoreceptors initiate downstream signal propagation that results in transient or sustained physiological responses (Gyula et al., 2003). In light signal transduction, CONSTITUTIVE PHOTOMORPHOGENIC1 (COP1) (Osterlund et al., 2000; Li et al., 2012), suppressor of phyA (SPA1) (Zuo et al., 2011), DE-ETIOLATED 1 (DET1) (Yanagawa et al., 2004), and PHYTOCHROME KINASE SUBSTRATE 1 (PKS1) (Gyula et al., 2003) participate in light-induced plant development.

In the present study, four RNA samples before and after light induction were sequenced by using the latest Illumina deep sequencing technique to elucidate light-induced anthocyanin accumulation in litchi pericarp. This study aimed to explain the molecular mechanisms of light-induced anthocyanin biosynthesis and to establish a solid foundation of future molecular studies on the basis of high-throughput sequencing and expression data.

## Materials and Methods

### Plant Materials, Shading Treatment, and Anthocyanin Content Determination

**FIGURE 1:**
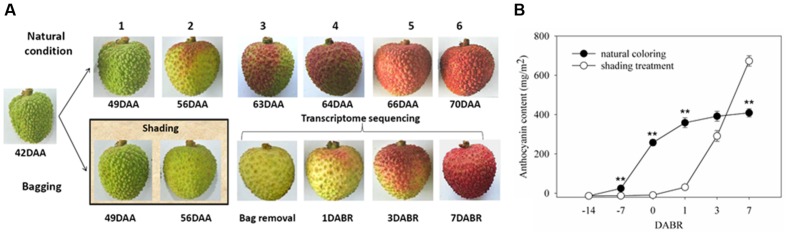
**Color and anthocyanin content of litchi pericarp. (A)** Images of litchi fruits in different coloration stages under natural conditions, shading and exposure. Samples corresponding to the developmental stages at 0, 1, 3, and 7 days after bags were removed were selected for transcriptome sequencing. **(B)** Total anthocyanin contents in the pericarp in different coloration stages. Vertical bars represent the standard error of three biological replicates. ‘^∗∗^’ represents significantly differences at the level of *P* < 0.01.

Samples were collected from 8-year-old litchi (*L. chinensis* Sonn. cv. Feizixiao) plants grown in an experimental orchard at the South Subtropical Crops Research Institute, Zhanjiang, Guangdong, China. Three trees were selected as biological replicates. Twenty clusters existing in different directions of the canopy were retained in each plant. Treatment was administered at 42 days after full bloom, while the color of pericarp absolutely unchanged red and the seed was entirely wrapped with pulp. Ten clusters were kept as natural coloring. The rest ten clusters were bagged with double-layer Kraft paper bags, and the bags were removed until the fruit of the control clusters coloring more than half (63 DAA) (**Figure 1A**). One individual fruit has been sampling from every fruit cluster at 0 (63 DAA), 1 (64 DAA), 3 (66 DAA), and 7 (70 DAA) days after the bags were removed (DABR), and pericarp disks were punched at the same place of fruit shoulder and immediately frozen in liquid nitrogen and stored at –80°C until further processing. The pericarp samples from 10 fruits in same tree were mixed to one sample. According to BBCH phenological description (Wei et al., 2013), fruit latter development and maturity involves six stages (**Figure 1A**). In anthocyanin content assay, pericarps were collected in the six periods. Total anthocyanin levels were measured in accordance with previously described methods (Wei et al., 2011). Three replicate extractions were prepared for each biological sample. Among these samples, the candidate samples of the pericarps at 0, 1, 3, and 7 DABR were subjected to transcriptome sequencing and expression analysis.

### RNA Extraction, Library Construction, and Transcriptome Sequencing

Four libraries (0, 1, 3, and 7 days) were designed for RNA-Seq to obtain a general overview of the pericarp transcriptome in response to light induction. Total RNA was extracted from pericarp tissues by using a Quick RNA isolation kit in accordance with the manufacturer’s instructions (Huayueyang, China). The RNA samples were digested with DNase I (TaKaRa, Japan) to remove potential genomic DNA and purified with RNase-free columns (Huayueyang, China). The concentration and quality of each sample were determined by using an Agilent 2100 bioanalyzer. Three biological replicates were used in the RNA-Seq experiments involving each sample. Four high-quality RNA-seq libraries were pooled by mixing equal quantities of RNA from the three biological replicates in each stage. Each library was then sequenced in an Illumina HiSeq 2500 system by using a single-end mode by the staff at the Beijing Genome Institute (Zhang et al., 2014). Each pool was sequenced once in consideration of its high replicability with relatively slight technical variations in RNA-Seq data (Zenoni et al., 2010; Liu et al., 2012).

### *De novo* Assembly and Functional Annotation

After raw reads were filtered to exclude low complexity reads, transcriptome *de novo* assembly was performed by using Trinity, a short-read assembling program (Grabherr et al., 2011). The resulting sequences obtained with Trinity were called unigenes. All of the generated unigene sequences were aligned by BLASTX (*E*-value < 10^–5^) to public protein databases. Functional annotations were obtained. The following public protein databases were considered: NCBI non-redundant protein (Nr) database^1^, Swiss-Prot protein database (Swiss-Prot) database^2^, Kyoto Encyclopedia of Genes and Genomes (KEGG) database^3^, and Clusters of Orthologous Groups (COGs) database^4^ (Kanehisa et al., 2008).

Unigene expression level was normalized by calculating read per kilobase per million (RPKM) (Mortazavi et al., 2008). Differential expression analysis was conducted using edgeR (Robinson et al., 2010). Differentially expressed unigenes (DEGs) q between treated and control samples were identified if False Discovery Rate ≤ 0.001, | log_2_|≥ 1, and *P*-value < 0.01. The DEGs were further subjected to Gene Ontology (GO) enrichment analysis and KEGG Pathway enrichment analysis to verify biological significance. GO and KEGG enrichment analyses were conducted according to a method similar to that described by Zhang et al. (2013), based on hypergeometric test. A FDR < 0.05 was used as the threshold to determine significant GO/KEGG enrichment of the gene sets. To understand the dynamic changes in absolute expression during pericarp coloration, we performed a hierarchical cluster analysis of expression patterns by using MultiExperiment Viewer (v4.8) (Eisen et al., 1998).

### Quantitative-PCR Analysis

Real-time quantitative reverse transcription-PCR (Q-PCR) was applied to investigate gene expression patterns. RNA was isolated from the samples in the four different coloring stages under light induction conditions and then used to generate cDNA. The list of gene-specific primers was shown in Additional file 1. Each reaction (final volume, 10 μL) contained 5 μL 2 × SYBR Green master mix (Thermo Fisher), 1 μL of each the forward and reverse primers (0.25 μM), 1 μL of the cDNA template (corresponding to 25 ng of total RNA), and 2 μL of RNase-free water. Q-PCR was conducted in a LightCycler^®^ 480 system (Roche, USA) under the following parameters: 95°C for 7 min and 40 cycles of 95°C for 10 s, 55°C for 15 s, and 72°C for 25 s. The unigene expression levels were calculated with the 2^–ΔΔCT^ method (Livak and Schmittgen, 2001). The litchi actin gene (GenBank Accession number HQ615689) was amplified as an internal control (Zhong et al., 2011). Q-PCR analyses were performed thrice with independent RNA samples.

## Results

### Rapid Anthocyanin Accumulation after Fruit Exposure

In this study, 10 clusters were bagged with double-layer Kraft paper bags at 42 days after anthesis (DAA) to simulate shaded conditions. These clusters were then exposed to light by removing the bags at 63 DAA when the control fruits color break. In **Figure 1A**, the litchi pericarp gradually turned red as the fruits matured under natural conditions; by contrast, the pericarp remained light green under shaded conditions and rapidly became red after the fruits were exposed to light. These phenomena were consistent with the changes in the abundance of anthocyanins in the pericarps. In general, anthocyanin contents gradually increase during natural coloration. In this study, anthocyanins were not detected in the pericarp under shaded conditions. After the fruits were exposed to light, the anthocyanin contents increased remarkably within 1 week (**Figure 1B**). These results suggested that light is essential for anthocyanin biosynthesis in litchi pericarp. However, the mechanism of light-induced anthocyanin biosynthesis remains unknown. To gain insights into this mechanism, we conducted a global mRNA sequencing of the samples at 0, 1, 3, and 7 DABR.

### Transcriptome Sequencing, Assembly, and Annotation

**FIGURE 2:**
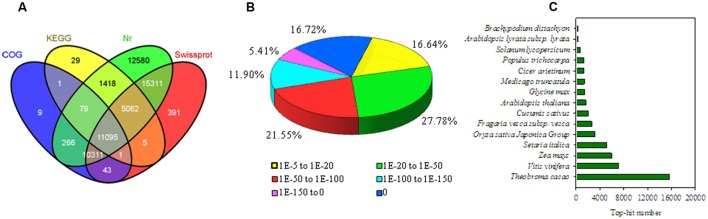
**Characteristics of the homology search of litchi unigenes. (A)** Venn diagram of the number of unigenes annotated against protein databases by BLASTing with an E-value threshold of 10^–5^. The numbers in the circles indicate the number of unigenes annotated by single or multiple databases. **(B)** *E*-value distribution of the top BLASTx hits for each unique sequence. **(C)** Species distribution of Nr annotation results of the homologous sequences.

Four libraries were constructed from the pericarps at 0, 1, 3, and 7 DABR. These libraries were sequenced with HiSeq 2000. The RNA-seq generated 19–22 million high-quality filtered reads, with approximately 2.0 billion nucleotides from each sample. The consensus assembly from the four samples generated 75,935 unigenes with an average length of 913 bp (**Table 1**). Among the assembled unigenes, approximately 74.5% (56,601) were annotated on the basis of BLASTx (*E*-value < 10^–5^) searches against the following public databases: Nr, Swiss-Prot, KEGG, GO database, and COG (**Figure 2A**), and 11,095 (14.6%) unigenes could be annotated to the all databases (**Figure 2A**). On the basis of *E*-value distribution, we found that approximately 34.0% of the annotated sequences showed very strong homology (*E*-value < 1e-100) to available plant sequences (**Figure 2B**). The Nr annotation revealed that 15,611 unigenes (27.9%) yielded top matches to sequences from *Theobroma cacao* (**Figure 2C**).

**Table 1:** Summary statistics of sequencing and assembly.

Samples (days)	Total reads	Total nucleotides (nt)	GC percentage	Total number of unigenes	Average length of unigenes (bp)
0	19,619,150	1,961,915,000	45.53%	37,782	928
1	22,795,142	2,279,514,200	45.71%	55,164	783
3	20,576,876	2,057,687,600	45.53%	40,258	981
7	21,620,592	2,162,059,200	45.18%	40,171	959
All	–	–	–	75,935	913

### Functional Classification

We used GO, COG, and KEGG assignments to classify the functions of the predicted unigenes of litchi. A total of 24,168 unigenes could be annotated using GO and classified on the basis of “biological process,” “cellular component,” and “molecular function” (Additional file 2). Interestingly, “cellular process” (13,639, 56.4%), “metabolic process” (13,445, 55.6%), “cell” (16,228, 67.2%), “cell part” (16,228, 67.2%), “catalytic activity” (12,217, 50.6%), and “binding” (11,386, 47.1%) were significantly overrepresented in the 43 GO subcategories (Additional file 2). Overall, 21,805 unigenes were categorized into COG functional groups. Among the 25 categories, “general function prediction only” (7,229, 17.2%) represented the largest group, “transcription” (4,181, 9.97%), and “replication recombination and repair” (3,611, 8.61%) were the next most common groups (Additional file 3). KEGG Pathway mapping indicated the possible biological interpretation of the assigned functions. This finding can enhance our understanding of the biological functions of genes. A total of 17,690 unigenes were mapped into 125 KEGG pathways (Additional file 4). The most represented pathways were “metabolic pathways” (4,562, 25.8%), followed by “biosynthesis of secondary metabolites” (2, 238, 12.7%).

### Differential Gene Expression during Light Induction

**FIGURE 3:**
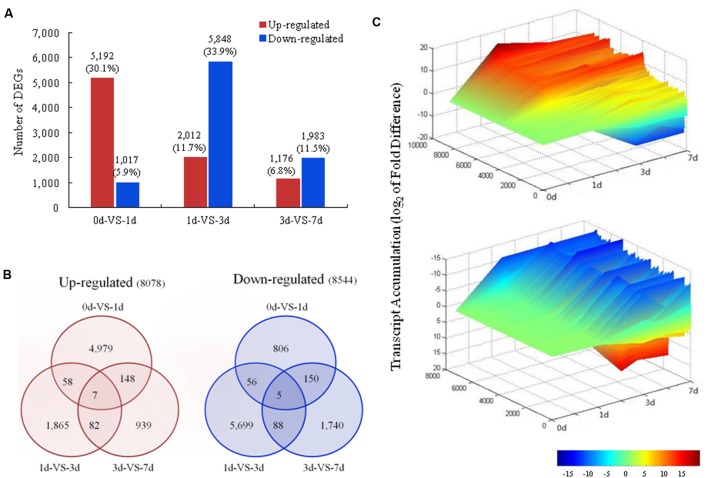
**Changes in gene expression profiles during light induction. (A)** The numbers of DEGs in pairwise comparisons of the four libraries; **(B)** Venn diagram showing the number of upregulated and downregulated genes revealed by paired comparison; **(C)** Overall expression profiles of the unigenes expressed in the libraries in four different stages.

Differences in gene expression during coloration were examined to identify the candidate genes involved in light-induced coloration. A total of 17,228 DEGs were identified during coloration through pairwise comparisons, with 6,209, 7,860, and 3,159 showing differential expression between 0 and 1 DABR, between 1 and 3 DABR, and between 3 and 7 DABR, respectively (**Figure 3A**). All 16,622 unigenes in the three pairwise comparisons were differentially expressed during coloration and were further analyzed (**Figure 3B**). For a global view of the expression patterns, the expression level of these unigenes was visualized in three-dimensional space (**Figure 3C**), which provides an overall understanding of the changes in unigene expression. The greatest number of DEGs (5,192; 30.1%) among the upregulated genes was found between 0 and 1 day libraries (**Figure 3A**). The relationship of DEGs among the different comparisons showed that 4,979 unigenes were increased in 1 day (**Figure 3B**). These findings suggested that most transcripts were transiently and significantly regulated after the fruits were exposed to light. By contrast, the greatest number of DEGs (5,848; 33.9%) among the downregulated genes was detected between 1 and 3 days libraries (**Figure 3A**). Of the 8,544 downregulated unigenes among 0, 1, 3, and 7 days libraries, 5,699 (66.7%) were decreased in 3 days library (**Figure 3B**). This finding indicated that the expression levels of the abundant genes decreased in the middle stage after the fruits were exposed to light.

The DEGs subjected to pairwise comparisons, namely, 0d-VS-1d, 1d-VS-3d, and 3d-VS-7d, were evaluated through GO and KEGG functional analyses to further predict the light-induced genes. The proportions and comparisons between DEGs were summarized in three main GO categories (Additional file 5). In the cellular component category, no GO terms were significantly enriched in the three pairwise comparisons. By contrast, “intracellular part” and “intracellular” were significantly enriched in the 1d-VS-3d comparison. Some GO terms, such as “photosystem,” “photosystem II,” “chloroplast part,” and “chloroplast thylakoid,” related to light reaction were significantly enriched in the 0d-VS-1d and 3d-VS-7d comparisons (Additional file 5A). In the molecular function category, one GO term, namely, “oxidoreductase activity,” was significantly enriched in the three pairwise comparisons. The number of “oxidoreductase activity” in 3d-VS-7d was significantly higher than those in 0d-VS-1d and 1d-VS-3d comparisons (Additional file 5B). In the biological process category, most biological processes, including “photosynthesis,” “light reaction,” “carbohydrate catabolic process,” “cellular biosynthetic process,” and “biosynthetic process,” were significantly enriched in 0d-VS-1d. “Cell wall biogenesis,” “cell wall organization,” “pyruvate metabolic process,” and “phenylpropanoid metabolic process” were significantly enriched in 3d-VS-7d (Additional file 5C).

In 0d-VS-1d, 1d-VS-3d, and 3d-VS-7d comparisons, 2,244, 2,756, and 721 DEGs were mapped to 118, 121, and 105 KEGG pathways, respectively. Of the 2,244 DEGs in 0d-VS-1d, 1,196 (53.3%) were mapped to 9 pathways (Additional file 6). The specific enrichment of the unigenes was observed in 9 pathways involved in metabolic processes, such as “photosynthesis,” “photosynthesis-antenna proteins,” “biosynthesis of secondary metabolites,” “flavonoid biosynthesis,” and “peroxisome.” Comparing 1 and 3 d libraries, we found that 334 (12.1%) DEGs were identified in 7 pathways (Additional file 6). In 3d-VS-7d, 625 (86.7%) DEGs were significantly enriched in 15 pathways, including “phenylpropanoid biosynthesis,” “photosynthesis,” “phenylalanine metabolism,” “metabolic pathways,” “biosynthesis of secondary metabolites,” “flavonoid biosynthesis,” “zeatin biosynthesis,” “starch and sucrose metabolism,” and “pentose and glucuronate interconversions” (Additional file 6).

### Changes in Gene Expression Profiles during Light Induction

**FIGURE 4:**
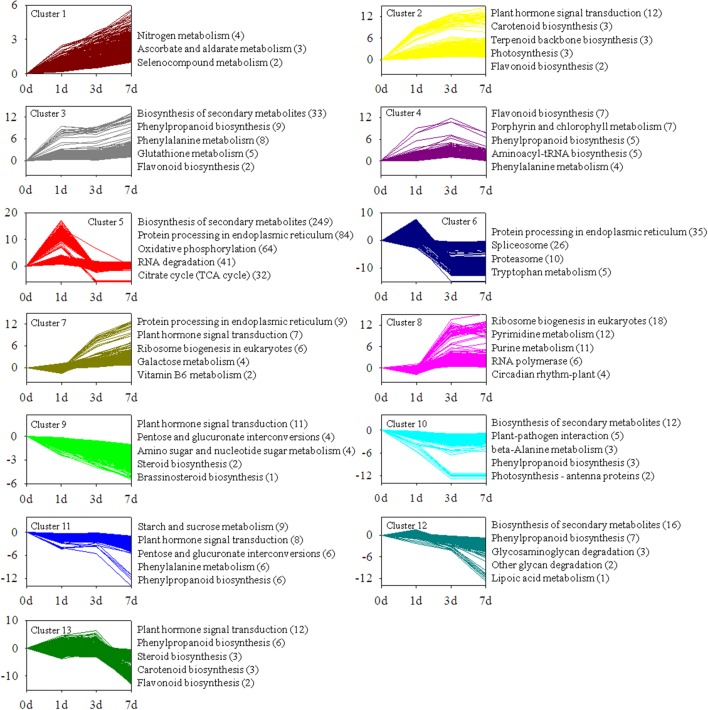
**Cluster analysis of differentially expressed transcripts with significant expression profile changes and KEGG pathway enrichment analysis.** Genes coding for unknown products were not considered in the analysis. Enriched KEGG pathways are listed to the right of each cluster.

The transcripts were divided into 25 clusters representing distinct expression patterns to gain further insights into the biological processes involved in light-induced responses by using Short Time-series Expression Miner software package (Additional file 7). **Figure 6** illustrates 13 clusters comprising 3,956 transcripts with significant differential expression at *P*-value < 0.05 (Additional file 7). To provide a global description of the biological pathway enriched in each cluster of similarly regulated transcripts, we also presented an overview of the KEGG pathway enrichment (**Figure 4**).

Clusters 1, 2, 3, and 4 contained genes positively modulated in the whole time course. These genes include those involved in “flavonoid biosynthesis,” “carotenoid biosynthesis,” “plant hormone signal transduction,” and “photosynthesis.” Clusters 5 and 6 contained genes upregulated at 1 d time point. This result suggested that these genes were induced in early stages of light induction. The genes specifically induced in late stages of light induction were grouped in clusters 7 and 8. The expression levels of the genes in clusters 9, 10, 11, 12 and 13, including a broad range of genes responsible for “plant hormone signal transduction,” “phenylpropanoid biosynthesis,” and “biosynthesis of secondary metabolites,” were significantly downregulated from 0 to 7 days.

### Plant Light Signal Perception and Transduction

**FIGURE 5:**
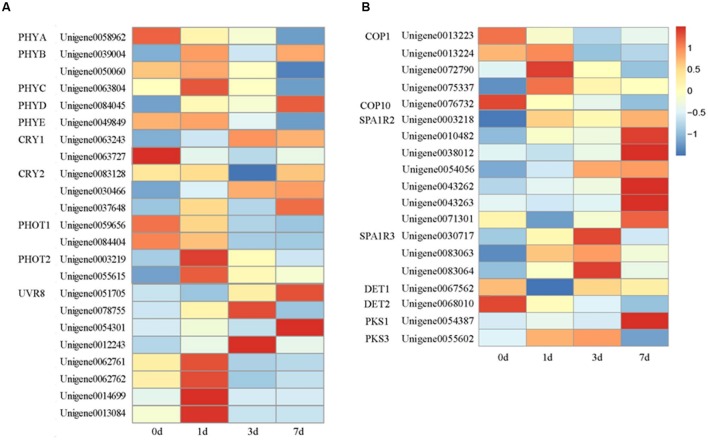
**Heat map representation of the expression patterns of photoreceptors (A) and genes related to light signal transduction (B).** PHYA, Phytochrome A; PHYB, Phytochrome B; PHYC, Phytochrome C; PHYD, Phytochrome D; PHYE, Phytochrome E; CRY1, Cryptochrome 1; CRY2, Cryptochrome 2; PHOT1, Phototropin 1; PHOT2, Phototropin 2; UVR8, UV RESISTANCE LOCUS 8; COP1, CONSTITUTIVE HOTOMORPHOGENIC 1; SPA1, Suppressor of phyA; DET1, DE-ETIOLATED 1; DET2, DE-ETIOLATED 2; PKS1, PHYTOCHROME KINASE SUBSTRATE 1; and PKS3, PHYTOCHROME KINASE SUBSTRATE 3. Columns and rows in the heat map represent the samples collected at different time points at which the bags were removed. The color scale at the right represents the log-transformed RPKM value.

Plant responses to light are extensive and are mediated by four different classes of photoreceptors: PHYs, CRYs (Chaves et al., 2011), PHOTs (Christie, 2007), and UVR8 (Jenkins, 2014). PHYs are encoded by a multigene family comprising five genes of the herbaceous *Arabidopsis thaliana* (PHYA to PHYE) (Opseth et al., 2015). In our study, the expression of putative *PHYA* and *PHYE* decreased after the bags were removed. By contrast, putative *PHYB* and *PHYC* reached their highest level at 1 DABR and then gradually decreased; putative *PHYD* peaked at 7 DABR (**Figure 5A**). Many plant species contain several CRY genes. CRY1, CRY2, and CRY3 (also denoted as CRY-DASH) have been isolated from *A. thaliana* (Opseth et al., 2015). Putative CRY1 and CRY2 have been identified in litchi. Two CRY1s, *Unigene 0063243* and *Unigene 0063727*, transcriptionally differed. The expression level of *Unigene 0063243* gradually increased after the bags were removed and then peaked at 3 DABR. By comparison, *Unigene 0063727* decreased after the fruits were exposed to light. The expression patterns of three CRY2s increased to varying degrees after the bags were removed and peaked at 7 DABR (**Figure 5A**).

UVR8 was first isolated from *A. thaliana* (Kliebenstein et al., 2002). This component acts as a UV-B photoreceptor (Rizzini et al., 2011). UVR8 mediates photomorphogenic responses to UV-B by regulating the transcription of a set of target genes (Jenkins, 2014). In our study, eight genes putatively encoding UVR8 were identified in litchi. All of their expression levels markedly increased after the bags were removed but peaked at different times. Among these genes, four responded promptly and reached the highest expression levels at 1 DABR, two peaked at 3 DABR, and two exhibited an increase in expression within the sampling period (**Figure 5A**).

Located downstream of the photoreceptors, ubiquitin E3 ligase COP1 acts as a molecular switch of light-induced plant development processes, such as photomorphogenesis (Hong et al., 2008), photoperiodic growth (Yu et al., 2008) and anthocyanin biosynthesis (Li et al., 2012). After the genes were separated and enriched, five genes putatively encoding COP1 were identified in litchi. After the fruits were exposed to light, the transcription levels of and one COP1 gene (*Unigene 0013223*) decreased until the fruits were fully matured. By contrast, the expression levels of the three other unigenes, namely, *Unigene 0013224*, *Unigene 0072790*, and *Unigene 0075337*, increased rapidly after the bags were removed and reached the highest level at 1 DABR. Afterward, their expression levels gradually decreased. Similar to the transcription level of *Unigene 0076732*, the transcription level of putative *COP10* gradually decreased after the fruits were exposed to light (**Figure 5B**).

SPA1, DET1, De-etiolated 2 (DET2), PHYTOCHROME KINASE SUBSTRATE1 (PKS1), and PKS3 are important elements in the light signaling pathway (Huang et al., 2014). In our study, the transcription level of putative *SPA1R2* reached the highest at 7 DABR, whereas the expression levels of putative *SPA1R3* peaked at 3 DABR. Moreover, the expression values of putative *DET1* and *DET2* decreased; by contrast, the expression levels of putative *PKS1* and *PKS3* gradually increased after the bags were removed (**Figure 5B**).

### Transcription Factors

**FIGURE 6:**
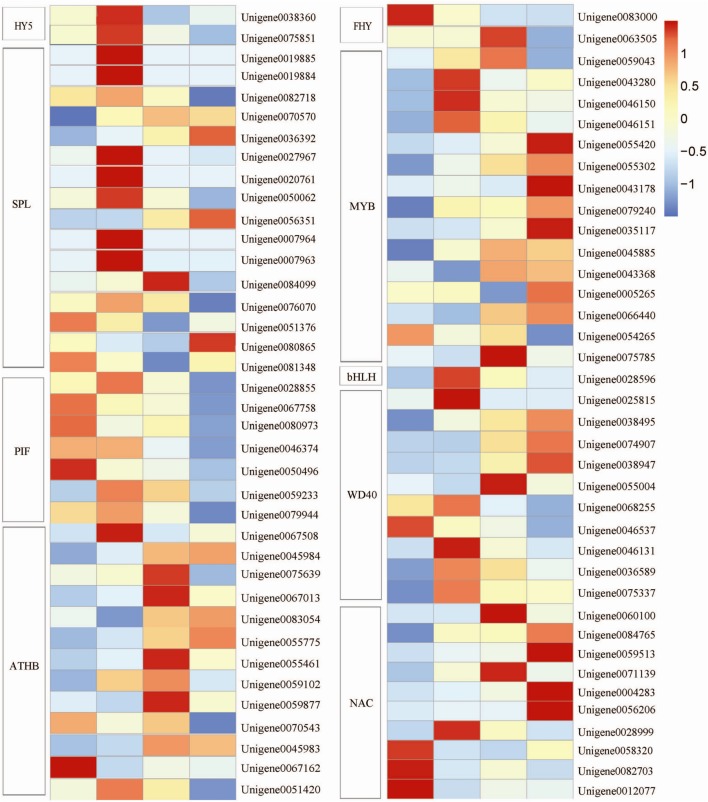
**Heat map representation of the expression patterns of light-related transcription factor (TF) genes.** *HY5*, ELONGATED HYPOCOTYL; *SPL*, SQUAMOSA promoter-binding protein-like; *PIF*, phytochrome-interacting factors; *ATHB*, homeodomain leucine zipper proteins; *FHY*, FAR-RED ELONGATED HYPOCOTYL; *bHLH*, basic helix-loop-helix; *WD40*, WD40 protein; *NAC*, NAM/ATAF/CUC. Columns and rows in the heat map represent the samples collected at different time points at which the bags were removed. The color scale at the right represents the log-transformed RPKM value.

Molecular genetic studies have demonstrated the crucial role of TFs in plant growth and development. In our study, 76 putative TFs which regulated anthocyanin biosynthesis and exhibited highly dynamic changes in response to light were identified. Among various TF families, SQUAMOSA promoter-binding protein-like (SPL) TF constituted the largest group (21.1%), followed by MYBs (19.7%), homeodomain leucine zipper proteins (ATHB, 17.1%), WD40 (13.2%), NAM/ATAF/CUC (NAC, 13.2%), phytochrome-interacting factors (PIFs, 9.2%), FAR-RED ELONGATED HYPOCOTYL (FHY3, 2.6%), ELONGATED HYPOCOTYL (HY5, 2.6%), and basic helix-loop-helix (bHLH, 1.3%) (**Figure 6**).

Among various complex TFs, the MBW (MYB-bHLH-WD40) transcription complex containing *MYB*, *bHLH*, and *WD40* is possibly the major determinant of anthocyanin biosynthesis (Allan et al., 2008; Hichri et al., 2011). In our study, 15 MYB-putatively encoding genes were identified. These genes except *Unigene 0054265* were upregulated after the fruits were exposed to light, but the peak transcriptional levels were detected at different time points. Furthermore, 10 putative *WD40* genes and 1 putative *bHLH* gene were observed. The expression levels of these genes were increased significantly. In addition, 10 putative NAC TFs were identified; of these 10 TFs, 7 were upregulated and 3 were downregulated after the bags were removed (**Figure 6**).

Other light-induced TFs, including putative *HY5*, *SPL*, *PIF*, *ATHB*, and *FHY*, were identified. The expression level of *HY5* reached the peak at 1 DABR; afterward, the expression level gradually declined. The expression levels of the SPLs except *Unigene 0051376* and *Unigene 0081348* were upregulated after the bags were removed. The seven identified *PIF*s showed a differential expression pattern after the bags were removed. The expression levels of four *PIF*s, *Unigene 0067758*, *Unigene 0080973*, *Unigene 0046374*, and *Unigene 0050496*, were the highest under shaded conditions. These levels gradually declined after the bags were removed. By contrast, the three other unigenes, *Unigene 0028855*, *Unigene 0059233*, and *Unigene 0079944*, reached the highest expression level at 1 DABR. The transcription levels of the *ATHB*s except *Unigene 0070543* and *Unigene 0067162* were upregulated. For two *FHY*s, the expression level of *Unigene 0083000* peaked before the bags were removed; by comparison, the expression level of *Unigene 0063505* reached the highest at 3 DABR (**Figure 6**).

### Structural Genes Involved in Anthocyanin Biosynthesis

**FIGURE 7:**
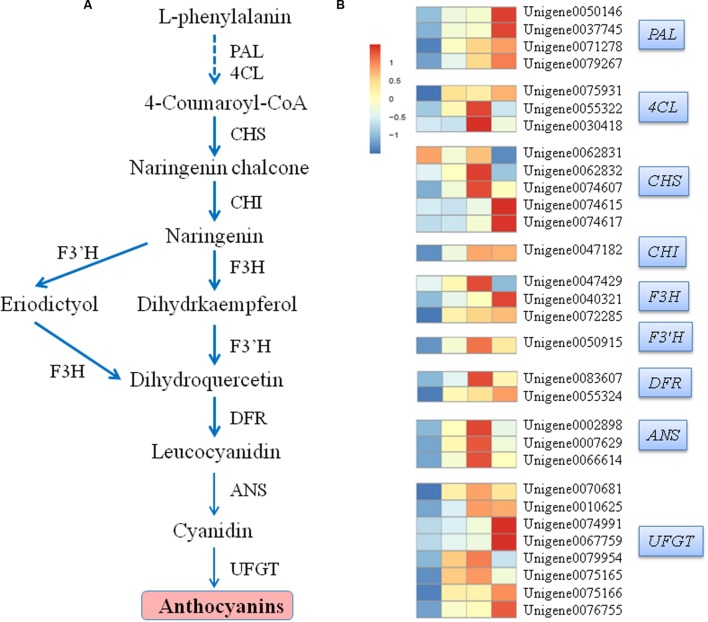
**Simplified scheme (A) and a heat map of the expression of genes (B) related to anthocyanin biosynthesis in litchi pericarp.** PAL, phenylalanine ammonia lyase; 4CL, 4-coumarate coenzyme A ligase; CHS, chalcone synthase; CHI, chalcone isomerase; F3H, flavanone 3-hydroxylase; F3′H, flavanone 3′-hydroxylase; DFR, dihydroflavonol-4-reductase; ANS, anthocyanidin synthase/leucoanthocyanidin dioxygenase; UFGT, UDP-glucose: flavonoid-3-*O*-glucosyltransferase. Enzyme names, unigene IDs, and expression patterns are indicated at the right side of each step.

Flavonoids are a diverse group of plant secondary metabolites with various biological functions during plant development. Flavonoid biosynthesis is a dynamic and complex process catalyzed by a series of enzymes (**Figure 7A**). In our study, 30 candidate transcripts were identified. These transcripts participated in each step of the anthocyanin metabolic pathway (**Figure 7B**). The transcriptional levels of structural genes, such as putative *PAL*, *4CL*, *CHS*, *CHI*, *F3H*, *F3*′*H*, *DFR*, *ANS*, and *UFGT*, increased in response to light exposure. The expression levels of the four identified phenylalanine ammonialyase (PAL) genes, *Unigene 0050146*, *Unigene 0037745*, *Unigene 0071278*, and *Unigene 0079267*, were the lowest when the pericarp was covered with a paper bag. By contrast, their expression levels gradually increased after the fruits were exposed to light. Three 4-coumarate coenzyme A ligase (4CL) genes were identified. Of these genes, *Unigene 0055322* and *Unigene 0030418* exhibited the highest expression levels at 3 DABR; *Unigene 0075931* reached the highest expression level at 7 DABR. Three distinct expression patterns were observed in the five chalcone synthase (CHS) genes. The transcription levels of the four *CHS* genes increased as the duration of exposure to light was prolonged. The transcription levels of *Unigene 0062832* and *Unigene 0074607* reached the highest at 3 DABR and those of *Unigene 0074615* and *Unigene 0074617* peaked at 7 DABR. By contrast, the expression level of *Unigene 0062831* was the highest before the bags were removed; after the bags were removed, its expression level decreased. Chalcone isomerase (*CHI*, *Unigene 0047182*), flavanone 3′-hydroxylase (*F3*′*H*, *Unigene 0050915*), and three anthocyanidin synthase/leucoanthocyanidin dioxygenase (*ANS*, *Unigene 0002898*, *Unigene 0007629*, and *Unigene 0066614*) genes displayed similar expression patterns, which were upregulated after the bags were removed, peaked at 3 DABR, and declined at 7 DABR. The expression levels of *Unigene 0040321* and *Unigene 0072285*, which encoding flavanone 3-hydroxylase (F3H), gradually increased as the duration of exposure to light was prolonged. Conversely, the expression level of *Unigene 0047429* (another *F3H* gene) reached the highest at 3 DABR (**Figure 7**).

UDP-glucose: flavonoid-3-*O*-glucosyltransferase plays a predominant role in anthocyanin accumulation and pericarp coloration in litchi (Wei et al., 2011). In our study, eight UFGT-putatively encoding genes were identified and subdivided into two distinct expression patterns. Furthermore, all unigenes were upregulated after the bags were removed. Among these genes, *Unigene 0070681*, *Unigene 0010625*, *Unigene 0079954*, and *Unigene 0075165*, reached the highest expression at 3 DABR; afterward, their expression levels decreased. By comparison, the other four genes, *Unigene 0074991*, *Unigene 0067759*, *Unigene 0075166*, and *Unigene 0076755*, peaked until 7 DABR (**Figure 7**).

### qRT-PCR Validation of Differentially Expressed Genes

**FIGURE 8:**
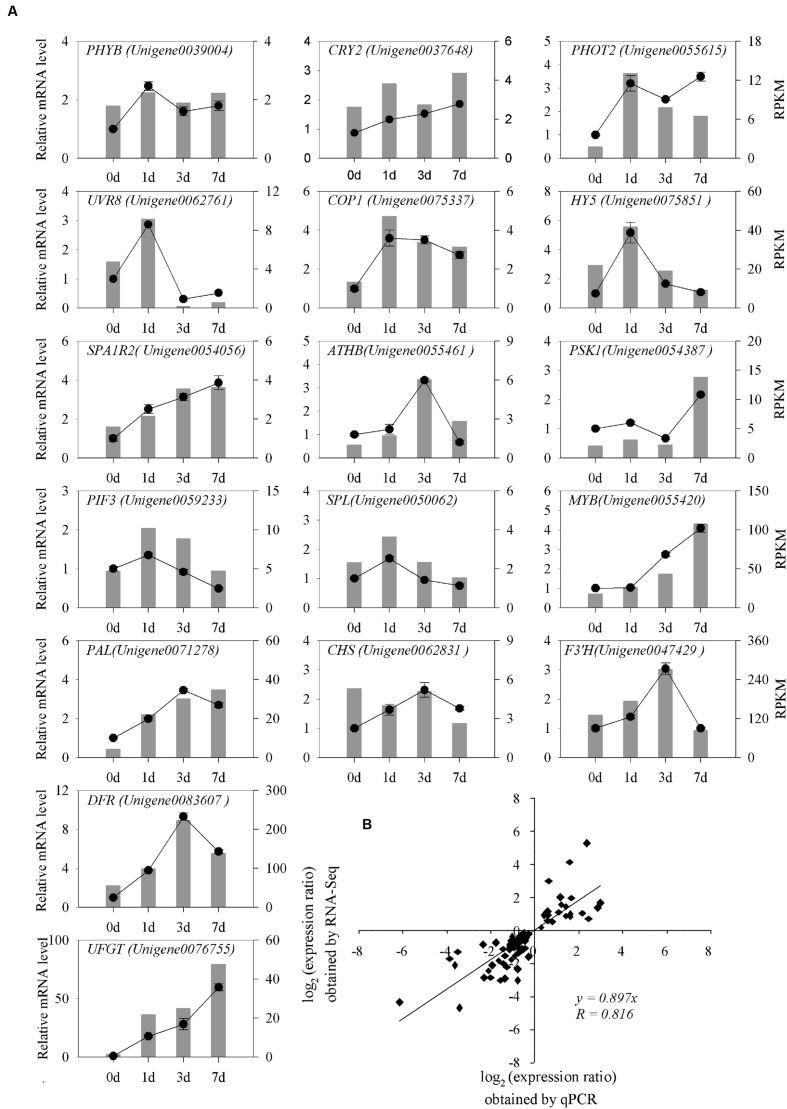
**qRT-PCR analysis of differentially expressed genes in litchi after the bags were removed. (A)** Transcript levels and qRT-PCR results of 17 randomly selected genes from RNA-sequencing. The left y-axis shows the relative gene expression levels analyzed by qPCR (black lines). The right y-axis indicates the corresponding expression data of RNA-seq (gray histogram). The x-axis represents the time (days) of light induction. Bars represent SE (*n* = 3). **(B)** Comparison between the log2 of the gene expression ratios obtained from RNA-seq data and qRT-PCR results.

To further validate the RNA-seq results, we selected 17 light-induced unigenes and subjected them to qRT-PCR analysis 0, 1, 3, and 7 DABR (**Figure 8A**). These unigenes involved in plant light signal perception and transduction, TFs, and structural genes in pathway of anthocyanin biosynthesis. In the four sampling stages, the qRT-PCR results were strongly correlated with the RNA-seq-generated data (Pearson correlation coefficients *R* = 0.816; **Figure 8B**). These results indicated that the transcriptomic profiling data accurately corresponded to light responses of litchi pericarp.

## Discussion

Light is a key environmental factor affecting anthocyanin biosynthesis in plants (Zoratti et al., 2014). In our study, shaded conditions inhibited anthocyanin accumulation in litchi pericarp. After the fruits were exposed to light, anthocyanins were rapidly synthesized within a short time (**Figure 1**). It can enhances the effect of anthocyanin biosynthesis and also effectively avoid other environmental factors influencing fruit coloration. This finding indicated that the coloration of litchi fruit depends on light, as observed in previous studies on litchi (Wei et al., 2011; Lai et al., 2014), grape berry (Jeong et al., 2004; Azuma et al., 2012), Chinese bayberry (Niu et al., 2010), apple (Li et al., 2012), and red pear (Steyn et al., 2004).

Plants use multiple sensory photoreceptors to accurately perceive light conditions ranging from UV-B to far-red wavelengths and thus coordinate their responses to environments with ambient light (Wagner et al., 2005; Möglich et al., 2010). Four main types of photoreceptors, including PHYs (PHYA, PHYB, PHYC, PHYD, and PHYE) absorbing red/far-red light, CRYs (CRY1, CRY2, and CRY3), PHOTs (PHOT1 and PHOT2) sensing UV-A/blue light, and UVR8 perceiving UV-B (Casal, 2013; Zoratti et al., 2014), have been identified. In our study, 23 light receptors, including six PHYs, five CRYs, four PHOTs, and eight UVR8s, were observed in the differentially enriched genes (**Figure 5**). After the bags were removed, the transcription levels of one PHYB, one PHYC, one PHOT2, and four UVR8s remarkably increased and reached the highest at 1 DABR paralleling with the upregulation of structural and regulation genes in anthocyanin biosynthesis pathway. These results indicated that PHYB, PHYC, PHOT2, and UVR8 might participate in the regulation of anthocyanin biosynthesis. Among these photoreceptors, UVR8s might be the most important in the pericarp of litchi. UV-B might elicit the strongest effect on the coloration of litchi pericarp. In *A. thaliana* and other fruit trees, the effect of UV-B promoting anthocyanin biosynthesis is more remarkable than that of blue right, white light, red light, and far-red light (Wang and Wang, 2004; Ubi et al., 2006; Zhang et al., 2012).

Under light conditions, photoreceptors receive light signals; signal transduction elements are then transduced to regulate light-dependent responses in plants (Zoratti et al., 2014). COP1 is a multifunctional RING E3 ubiquitin ligase that plays an important role in organ development, seedling photomorphogenesis, environmental stress responses, and crosstalk between light and phytohormone signaling pathways in *Arabidopsis* (Huang et al., 2014). Similarly, plant orthologs of COP1 regulate fruit coloration in apple (*Malus domestica*) (Li et al., 2012) and gibberellin biosynthesis in peas (*Pisum sativum*) (Weller et al., 2009). Under dark conditions, COP1 is localized in the nucleus and is associated with photomorphogenesis-promoting TFs, such as HY5 and MYB. When cells are exposed to light, the concentration of COP1 in the nucleus rapidly decreases (Hong et al., 2008). In our study, one of the four COP1s that were identified in litchi is consistent with that described in previous studies of *A. thaliana* and apple; thus, COP1 possibly exhibits a different pattern of light-dependent response regulation and gene expression. In addition, COP10 is a ubiquitin-conjugating enzyme variant, which is a negative regulator of photomorphogenesis essential for COP1-mediated HY5 degradation (Osterlund et al., 2000; Yanagawa et al., 2004). In litchi, the transcriptional level of COP10 gradually decreased after the bags were removed. This result is consistent with that observed in other plants.

The RING-finger domain of the COP1 protein can undergo self-ubiquitination; this domain can also ubiquitinate the TFs downstream (Kang et al., 2009). Thus, TFs activate the transcription of downstream genes in plants to stimulate photomorphogenesis (Henriques et al., 2009). MYB is a crucial regulator of light-induced anthocyanin accumulation and fruit coloration in several fruit crops, such as grape (Kobayashi et al., 2002), apple (Li et al., 2012), Chinese bayberry (Niu et al., 2010), and litchi (Lai et al., 2014). As such, MYB is implicated in blue-light response regulation (Li et al., 2012). Among the TFs identified in our study, the MBW transcription complex containing MYB, bHLH, and WD40 accounted for nearly one-third of the total number of TFs. The expressions were mostly upregulated when the litchi fruits were exposed to light. These results suggested that MBW could be involved in light-induced anthocyanin biosynthesis. In addition, *PpNAC1* can activate the transcription of *PpMYB10.1* (Zhou et al., 2015) to promote anthocyanins accumulate in peach, and the *PpNAC1* and *PpMYB10.1* expression in fruits is low in early developmental stages (Zhou et al., 2015). In our study, 10 NACs were identified in litchi. Of these NACs, 7 exhibited an increase in expression level when the fruits were exposed to light. The co-upregulation of MYB and NACs suggested that the activation of MYB might be associated with the removal of a transcriptional repressor. The expression levels of the three other NACs were higher under dark conditions than under light conditions. Furthermore, their expression levels decreased sharply after the fruits were exposed to light. This finding revealed that NAC may negatively regulate light-related responses in litchi.

As another important modulator of light signal coordination, HY5 positively regulates anthocyanin accumulation by directly binding to the promoters of structural genes in the anthocyanin biosynthetic pathway (Lee et al., 2007). Promoter analysis revealed that multiple light responsive *cis*-acting elements are present in MdHY5 promoters involved G-box, GT-1-box, and I-box (Li et al., 2014). In *A. thaliana*, SPL9 can negatively regulate anthocyanin accumulation by directly preventing structural gene expression in the anthocyanin biosynthetic pathway through the destabilization of an MBW transcriptional activation complex (Gou et al., 2011). Chromatin immunoprecipitation-quantitative PCR, electrophoretic mobility shift assay, and transient expression assays revealed that *PIF4* and *PIF5* can repress red light-induced promoter activities of *F3*′*H* and *DFR*; *PIF4* and *PIF5* can also negatively regulate red light-induced anthocyanin accumulation in *Arabidopsis* (Liu et al., 2015).

In our study, the transcription levels of HY5 rapidly increased after the bags were removed. This finding indicated that HY5 might be a positive regulator and be implicated in litchi fruit coloration. By contrast, the expression of most PIFs gradually declined as the fruits colored. These patterns are consistent with those observed in *Arabidopsis*. However, SPLs were identified as the most abundant TFs. Their expressions were upregulated after the fruits were exposed to light. This finding suggested that SPLs may play different roles in litchi when comparing with in *Arabidopsis*. In addition, 13 ATHBs and 2 FHYs were identified in this study. Although evidence has yet to be obtained to support that they directly regulated anthocyanin biosynthesis, they possibly participate in light signal transduction or other light-induced responses.

Fruit bagging and shading treatments have confirmed that nearly all structural genes in the anthocyanin biosynthetic pathway are influenced or regulated by light (Jeong et al., 2004; Steyn et al., 2004; Niu et al., 2010; Wei et al., 2011; Azuma et al., 2012). In our study, the transcriptional levels of many structural genes involved in the anthocyanin biosynthetic pathway increased significantly when the litchi fruits were exposed to light. These findings are consistent with those described in our previous studies (Wei et al., 2011; Lai et al., 2014).

**FIGURE 9:**
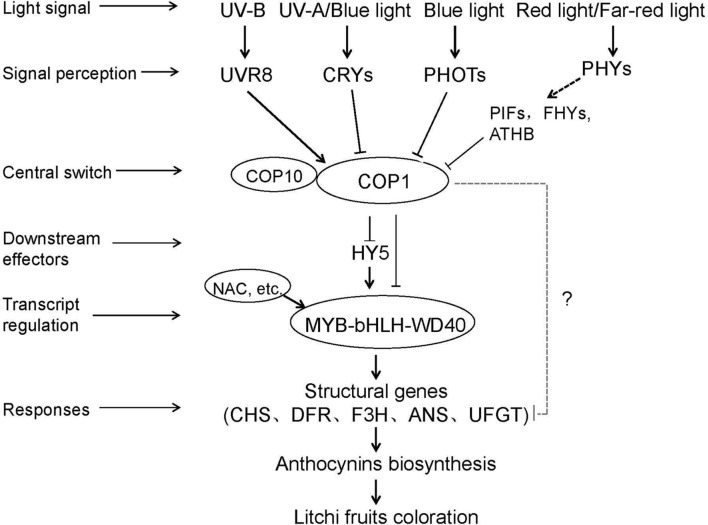
**A simplified model of the light-induced anthocyanin biosynthesis in litchi pericarp.** Firstly photoreceptors (e.g., UVR8, CRYs, and PHOTs) perceived light signal and then interact with COP1 to get rid of inhibiting effect of TFs (e.g., MBW and HY5). Soon after MBW complexes induced the transcription of structural genes (e.g., *LcUFGT*, *LcCHS*, *and LcDFR*). Finally, the activation and expression of *LcUFGT* promoted anthocyanin accumulation and thus stimulate the red coloration of litchi fruit.

On the basis of our research and previous studies involving model plants and other fruit trees (Lau and Deng, 2012; Jaakola, 2013; Zoratti et al., 2014), we summarized a simplified model of light-regulated anthocyanin biosynthesis (**Figure 9**). The light-induced regulation of anthocyanin biosynthesis is facilitated by photoreceptors, such as UVR8, CRYs, and PHYs, which perceive light signals with different wavelengths and then interact with COP1, which is a negative regulator that mediates the degradation of multiple light-response effectors, including HY5. Alternatively, COP1 can interact directly with MYB-bHLH-WD40 TF complexes to induce the transcription of structural genes, such as *LcUFGT*. The activation and expression of *LcUFGT* promote anthocyanin accumulation and thus stimulate the red coloration of litchi fruit. Nevertheless, whether or not COP1 immediately interacts with structural genes related to anthocyanin biosynthesis remains unknown.

## Conclusion

In our study, four sets of transcriptome data comprising 75,935 unigenes were generated in litchi pericarp by performing Illumina sequencing. A total of 16,622 different unigenes were identified on the basis of the dynamic changes in gene expression. These light-responsive genes encode photoreceptors, light signal transduction elements, TFs, and structural genes involved in the anthocyanin biosynthetic pathway. The transcriptome data and DEGs provided valuable information and gene sequences to investigate the regulatory mechanism of the coloration of litchi and other fruits.

### Availability of Supporting Data

We have deposit our data in Sequence Read Archive (SRA) ( http://www.ncbi.nlm.nih.gov/sra/), the accessions for our submission are: SRA312830. The raw data for the DGE analysis were also deposited in the NCBI Sequence Read Archive under accession numbers SRR2952606 (0 DABR), SRR2954687 (1 DABR), SRR2954690 (3 DABR), and SRR2954691 (7 DABR) (http://www.ncbi.nlm.nih.gov/sra/).

## Author Contributions

H-NZ performed most of the experiments and data analysis. Y-ZW and SYS conducted bioinformatics analyses and data interpretation. L-QL and BS carried out part of material collection, RNA extraction and data analysis. Y-ZW and W-CL participated in the preparation of the manuscript. J-HX and H-CW conceived, designed and coordinated the studies. All authors have read and approved the final manuscript.

## Conflictof Interest Statement

The authors declare that the research was conducted in the absence of any commercial or financial relationships that could be construed as a potential conflict of interest.

## Footnotes

1
http://www.ncbi.nlm.nih.gov

2
http://www.expasy.ch/sprot

3
http://www.genome.jp/kegg

4
http://www.ncbi.nlm.nih.gov/COG
