# Supplementary material for: Transcriptome Profiling of Light-Regulated Anthocyanin Biosynthesis in the Pericarp of Litchi
Source: Front Plant Sci. 2016 Jun 29;7:963. doi: 10.3389/fpls.2016.00963 (PMC4925703; doi:10.3389/fpls.2016.00963)
Supplement: Supplementary file 1 [file Data_Sheet_1.DOCX]

Supplementary Material

**Transcriptome profiling of light****-regulated anthocyanin biosynthesis in the pericarp of litchi**

**Hongna Zhang^1^, Weicai Li^1^, Huicong Wang^2^, Shengyou Shi^1^, Bo Shu^1^, Liqin Liu^1^, Yongzan Wei^1*^ and Jianghui Xie^1*^**

^1^ Key Laboratory of Tropical Fruit Biology (Ministry of Agriculture), South Subtropical Crops Research Institute, Chinese Academy of Tropical Agricultural Sciences, Zhanjiang, China.

^2^ College of Horticulture, South China Agricultural University, Guangzhou, China.

***Correspondence:** Y.Z. Wei and J.H. Xie, Key Laboratory of Tropical Fruit Biology (Ministry of Agriculture), South Subtropical Crops Research Institute, Chinese Academy of Tropical Agricultural Sciences, Zhanjiang, 524091, China.

**E-mail:** wyz4626@163.com; 2453880045@qq.com.

**Additional file 1**. The primer sequences of target and reference genes used in RT-qPCR analysis

|  | **Gene ID** | **Gene description** | **Forward primer (5' to 3')** | **Reverse primer (5' to 3')** |
| --- | --- | --- | --- | --- |
| 1 | Unigene0039004 | Phytochrome B | CCGACAGAAGGATGGGTAGAG | CCGTGGCATAGGAAGTTCAAG |
| 2 | Unigene0037648 | Cryptochrome 2 | GTGTCAAGGTGGTGGCTAAAG | ACAGGTTCCATTGGCATTTCC |
| 3 | Unigene0055615 | Phototropin 2 | ACCGATGCGTGACCAGAAG | GACAGGCTCAGAGTGAATTGC |
| 4 | Unigene0062761 | UVR8 | CCACGCTCTCAAGGTTCAAG | GCAATTCTGACTCCTGGGTTC |
| 5 | Unigene0075337 | CONSTITUTIVE HOTOMORPHOGENIC 1 | GTTCAGTGGGCACAGGAAAG | ATGGCACGACTTCATCTTCAG |
| 6 | Unigene0075851 | ELONGATED HYPOCOTYL | AGAACCAGCCCGAACATCAG | GCATCGCTACTACCTCCTCTC |
| 7 | Unigene0054056 | Suppressor of phyA | GTCTGTGGCTACTTCATCCTG | TCTGAACTTGACGGCTGTATG |
| 8 | Unigene0055461 | Homedomain leucine zipper proteins | AAGCAGATTGAGCACGAGTAC | GTTGTCAGTGTGGTCCAGTTC |
| 9 | Unigene0054387 | PHYTOCHROME KINASE SUBSTRATE 1 | TGTTTGGTTGCCCTGTGTTG | GGTTGCCTTTCGCTGTGAG |
| 10 | Unigene0059233 | Phytochrome interacting factors | TCGCAGGAGCAAGACTTGG | GCTTCGGATTGGTCATCTGG |
| 11 | Unigene0050062 | SQUAMOSA promoter-binding protein-like | TGAGGCTGGTGGTTCTAAGTG | AATGGTGGTTGACTCGTTACG |
| 12 | Unigene0055420 | MYB transcription factor | AGGCATCAGGCGAAGCAG | GGCGAAGTTGTGGTGGTATG |
| 13 | Unigene0071278 | Phenylalanine ammonia-lyase | CGGCTTGGCTTCTATGGTTC | TGCTTCGGCTTCTGGAGAG |
| 14 | Unigene0062831 | Chalcone synthase | CTACTTCCGAGTCACCAACAG | GATGCTGCTTCCTTGCCTAG |
| 15 | Unigene0047429 | Flavonoid 3'-hydroxylase | CACAGCCTGATTATCGTCTCC | GTCAACCCACTCAACCTTCTC |
| 16 | Unigene0083607 | Dihydroflavonol 4-reductase | GCTGGAGTGATATGGGCTTTG | TGGTGGCTGATTCTGAGGAG |
| 17 | Unigene0076755 | UDP-glucose: flavonoid 3-Oglucosyltransferase | TCGTGCCTTGTTGGTTATCAG | GCGTCTCCTTGTGGTAGTTG |
| 18 | HQ615689 | Actin | GTGGTTCTACTATGTTCCCTG | CTCGTCGTACTCATCCTTTG |


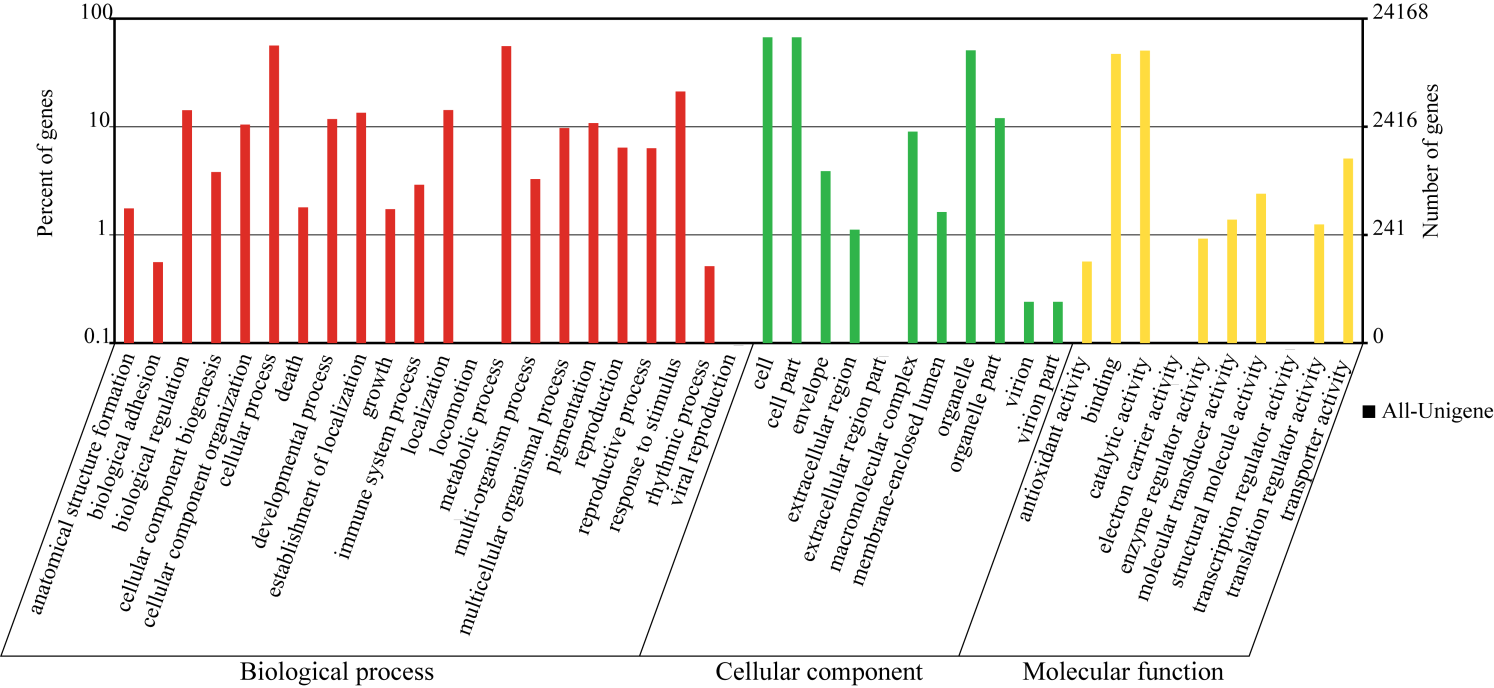


**Additional file 2**. Gene Ontology (GO) categories of the assembled litchi pericarp unigenes. Unigenes were assigned to three main categories: “biological process,” “cellular component,” and “molecular function.” The left- and right-side y-axes indicate the percentage and number of the annotated unigenes, respectively.


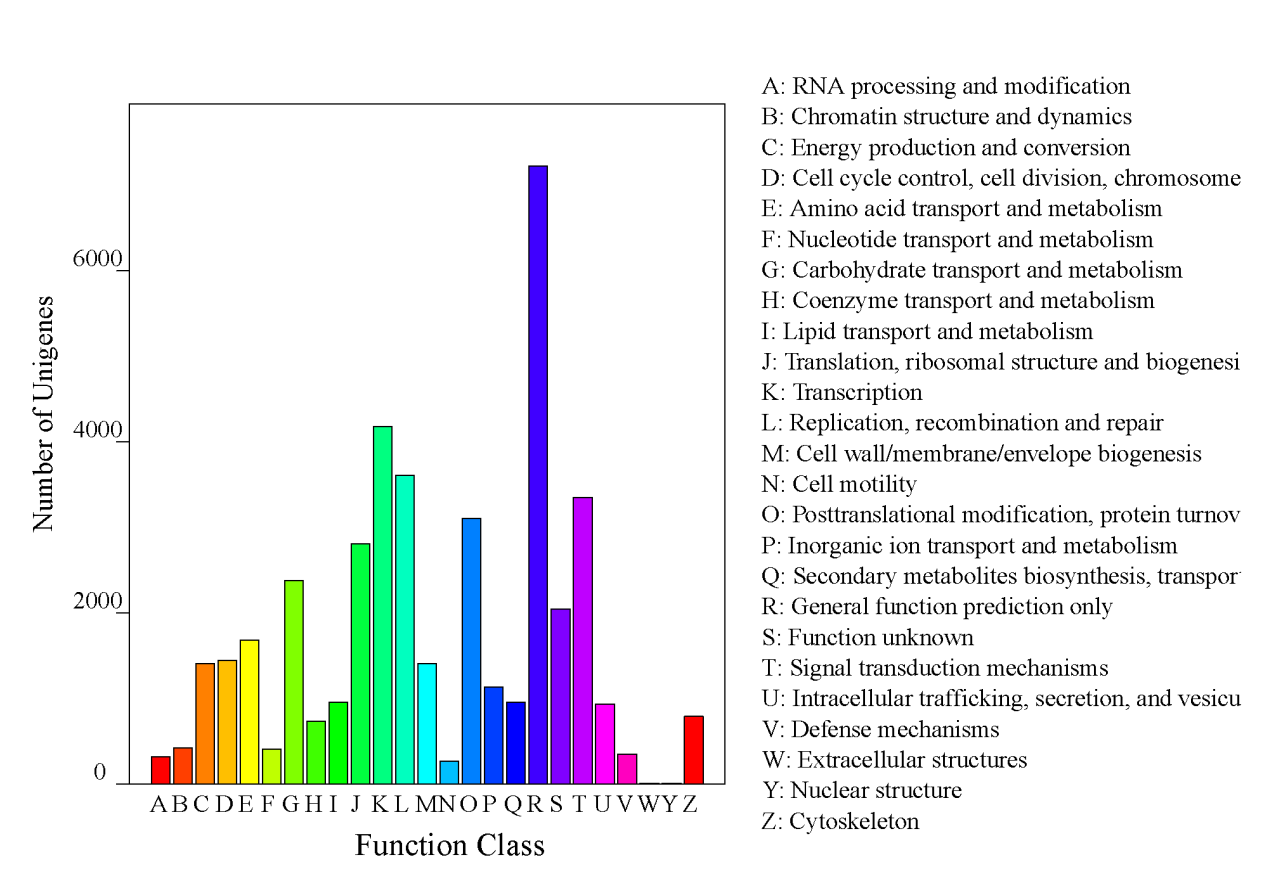


**Additional file 3**. COG functional classifications of the litchi pericarp transcriptome. A total of 21,805 unigenes were classified into 25 COG categories.

**Additional file 4.** KEGG pathways for all unigenes.

|  | **Pathway** | **All genes with pathway annotation (17690)** | **Pathway ID** |
| --- | --- | --- | --- |
| 1 | [Metabolic pathways](file:///H:\其他人转录组数据\魏永赞转录组\新数据\新数据\果皮转录组\附件\4-kegg-all%20genes.xlsx#RANGE!gene1) | 4562 (25.79%) | ko01100 |
| 2 | [Biosynthesis of secondary metabolites](file:///H:\其他人转录组数据\魏永赞转录组\新数据\新数据\果皮转录组\附件\4-kegg-all%20genes.xlsx#RANGE!gene2) | 2238 (12.65%) | ko01110 |
| 3 | [Ribosome](file:///H:\其他人转录组数据\魏永赞转录组\新数据\新数据\果皮转录组\附件\4-kegg-all%20genes.xlsx#RANGE!gene3) | 958 (5.42%) | ko03010 |
| 4 | [Spliceosome](file:///H:\其他人转录组数据\魏永赞转录组\新数据\新数据\果皮转录组\附件\4-kegg-all%20genes.xlsx#RANGE!gene4) | 629 (3.56%) | ko03040 |
| 5 | [RNA transport](file:///H:\其他人转录组数据\魏永赞转录组\新数据\新数据\果皮转录组\附件\4-kegg-all%20genes.xlsx#RANGE!gene5) | 616 (3.48%) | ko03013 |
| 6 | [Protein processing in endoplasmic reticulum](file:///H:\其他人转录组数据\魏永赞转录组\新数据\新数据\果皮转录组\附件\4-kegg-all%20genes.xlsx#RANGE!gene6) | 580 (3.28%) | ko04141 |
| 7 | [Oxidative phosphorylation](file:///H:\其他人转录组数据\魏永赞转录组\新数据\新数据\果皮转录组\附件\4-kegg-all%20genes.xlsx#RANGE!gene7) | 515 (2.91%) | ko00190 |
| 8 | [Plant-pathogen interaction](file:///H:\其他人转录组数据\魏永赞转录组\新数据\新数据\果皮转录组\附件\4-kegg-all%20genes.xlsx#RANGE!gene8) | 507 (2.87%) | ko04626 |
| 9 | [Plant hormone signal transduction](file:///H:\其他人转录组数据\魏永赞转录组\新数据\新数据\果皮转录组\附件\4-kegg-all%20genes.xlsx#RANGE!gene9) | 476 (2.69%) | ko04075 |
| 10 | [Ubiquitin mediated proteolysis](file:///H:\其他人转录组数据\魏永赞转录组\新数据\新数据\果皮转录组\附件\4-kegg-all%20genes.xlsx#RANGE!gene10) | 433 (2.45%) | ko04120 |
| 11 | [Glycolysis / Gluconeogenesis](file:///H:\其他人转录组数据\魏永赞转录组\新数据\新数据\果皮转录组\附件\4-kegg-all%20genes.xlsx#RANGE!gene11) | 419 (2.37%) | ko00010 |
| 12 | [Purine metabolism](file:///H:\其他人转录组数据\魏永赞转录组\新数据\新数据\果皮转录组\附件\4-kegg-all%20genes.xlsx#RANGE!gene12) | 416 (2.35%) | ko00230 |
| 13 | [mRNA surveillance pathway](file:///H:\其他人转录组数据\魏永赞转录组\新数据\新数据\果皮转录组\附件\4-kegg-all%20genes.xlsx#RANGE!gene13) | 397 (2.24%) | ko03015 |
| 14 | [Starch and sucrose metabolism](file:///H:\其他人转录组数据\魏永赞转录组\新数据\新数据\果皮转录组\附件\4-kegg-all%20genes.xlsx#RANGE!gene14) | 368 (2.08%) | ko00500 |
| 15 | [Ribosome biogenesis in eukaryotes](file:///H:\其他人转录组数据\魏永赞转录组\新数据\新数据\果皮转录组\附件\4-kegg-all%20genes.xlsx#RANGE!gene15) | 329 (1.86%) | ko03008 |
| 16 | [Endocytosis](file:///H:\其他人转录组数据\魏永赞转录组\新数据\新数据\果皮转录组\附件\4-kegg-all%20genes.xlsx#RANGE!gene16) | 322 (1.82%) | ko04144 |
| 17 | [Phagosome](file:///H:\其他人转录组数据\魏永赞转录组\新数据\新数据\果皮转录组\附件\4-kegg-all%20genes.xlsx#RANGE!gene17) | 312 (1.76%) | ko04145 |
| 18 | [Pyrimidine metabolism](file:///H:\其他人转录组数据\魏永赞转录组\新数据\新数据\果皮转录组\附件\4-kegg-all%20genes.xlsx#RANGE!gene18) | 299 (1.69%) | ko00240 |
| 19 | [Carbon fixation in photosynthetic organisms](file:///H:\其他人转录组数据\魏永赞转录组\新数据\新数据\果皮转录组\附件\4-kegg-all%20genes.xlsx#RANGE!gene19) | 287 (1.62%) | ko00710 |
| 20 | [Amino sugar and nucleotide sugar metabolism](file:///H:\其他人转录组数据\魏永赞转录组\新数据\新数据\果皮转录组\附件\4-kegg-all%20genes.xlsx#RANGE!gene20) | 278 (1.57%) | ko00520 |
| 21 | [Pyruvate metabolism](file:///H:\其他人转录组数据\魏永赞转录组\新数据\新数据\果皮转录组\附件\4-kegg-all%20genes.xlsx#RANGE!gene21) | 271 (1.53%) | ko00620 |
| 22 | [RNA degradation](file:///H:\其他人转录组数据\魏永赞转录组\新数据\新数据\果皮转录组\附件\4-kegg-all%20genes.xlsx#RANGE!gene22) | 266 (1.5%) | ko03018 |
| 23 | [Arginine and proline metabolism](file:///H:\其他人转录组数据\魏永赞转录组\新数据\新数据\果皮转录组\附件\4-kegg-all%20genes.xlsx#RANGE!gene23) | 241 (1.36%) | ko00330 |
| 24 | [Cysteine and methionine metabolism](file:///H:\其他人转录组数据\魏永赞转录组\新数据\新数据\果皮转录组\附件\4-kegg-all%20genes.xlsx#RANGE!gene24) | 236 (1.33%) | ko00270 |
| 25 | [Citrate cycle (TCA cycle)](file:///H:\其他人转录组数据\魏永赞转录组\新数据\新数据\果皮转录组\附件\4-kegg-all%20genes.xlsx#RANGE!gene25) | 235 (1.33%) | ko00020 |
| 26 | [Peroxisome](file:///H:\其他人转录组数据\魏永赞转录组\新数据\新数据\果皮转录组\附件\4-kegg-all%20genes.xlsx#RANGE!gene26) | 230 (1.3%) | ko04146 |
| 27 | [Glycerophospholipid metabolism](file:///H:\其他人转录组数据\魏永赞转录组\新数据\新数据\果皮转录组\附件\4-kegg-all%20genes.xlsx#RANGE!gene27) | 206 (1.16%) | ko00564 |
| 28 | [Glyoxylate and dicarboxylate metabolism](file:///H:\其他人转录组数据\魏永赞转录组\新数据\新数据\果皮转录组\附件\4-kegg-all%20genes.xlsx#RANGE!gene28) | 200 (1.13%) | ko00630 |
| 29 | [Fructose and mannose metabolism](file:///H:\其他人转录组数据\魏永赞转录组\新数据\新数据\果皮转录组\附件\4-kegg-all%20genes.xlsx#RANGE!gene29) | 197 (1.11%) | ko00051 |
| 30 | [Pentose phosphate pathway](file:///H:\其他人转录组数据\魏永赞转录组\新数据\新数据\果皮转录组\附件\4-kegg-all%20genes.xlsx#RANGE!gene30) | 186 (1.05%) | ko00030 |
| 31 | [Proteasome](file:///H:\其他人转录组数据\魏永赞转录组\新数据\新数据\果皮转录组\附件\4-kegg-all%20genes.xlsx#RANGE!gene31) | 182 (1.03%) | ko03050 |
| 32 | [Glutathione metabolism](file:///H:\其他人转录组数据\魏永赞转录组\新数据\新数据\果皮转录组\附件\4-kegg-all%20genes.xlsx#RANGE!gene32) | 181 (1.02%) | ko00480 |
| 33 | [Aminoacyl-tRNA biosynthesis](file:///H:\其他人转录组数据\魏永赞转录组\新数据\新数据\果皮转录组\附件\4-kegg-all%20genes.xlsx#RANGE!gene33) | 175 (0.99%) | ko00970 |
| 34 | [Galactose metabolism](file:///H:\其他人转录组数据\魏永赞转录组\新数据\新数据\果皮转录组\附件\4-kegg-all%20genes.xlsx#RANGE!gene34) | 172 (0.97%) | ko00052 |
| 35 | [Alanine, aspartate and glutamate metabolism](file:///H:\其他人转录组数据\魏永赞转录组\新数据\新数据\果皮转录组\附件\4-kegg-all%20genes.xlsx#RANGE!gene35) | 167 (0.94%) | ko00250 |
| 36 | [Valine, leucine and isoleucine degradation](file:///H:\其他人转录组数据\魏永赞转录组\新数据\新数据\果皮转录组\附件\4-kegg-all%20genes.xlsx#RANGE!gene36) | 167 (0.94%) | ko00280 |
| 37 | [Inositol phosphate metabolism](file:///H:\其他人转录组数据\魏永赞转录组\新数据\新数据\果皮转录组\附件\4-kegg-all%20genes.xlsx#RANGE!gene37) | 158 (0.89%) | ko00562 |
| 38 | [Phenylpropanoid biosynthesis](file:///H:\其他人转录组数据\魏永赞转录组\新数据\新数据\果皮转录组\附件\4-kegg-all%20genes.xlsx#RANGE!gene38) | 156 (0.88%) | ko00940 |
| 39 | [Nucleotide excision repair](file:///H:\其他人转录组数据\魏永赞转录组\新数据\新数据\果皮转录组\附件\4-kegg-all%20genes.xlsx#RANGE!gene39) | 155 (0.88%) | ko03420 |
| 40 | [Phenylalanine metabolism](file:///H:\其他人转录组数据\魏永赞转录组\新数据\新数据\果皮转录组\附件\4-kegg-all%20genes.xlsx#RANGE!gene40) | 153 (0.86%) | ko00360 |
| 41 | [Phosphatidylinositol signaling system](file:///H:\其他人转录组数据\魏永赞转录组\新数据\新数据\果皮转录组\附件\4-kegg-all%20genes.xlsx#RANGE!gene41) | 151 (0.85%) | ko04070 |
| 42 | [Glycine, serine and threonine metabolism](file:///H:\其他人转录组数据\魏永赞转录组\新数据\新数据\果皮转录组\附件\4-kegg-all%20genes.xlsx#RANGE!gene42) | 141 (0.8%) | ko00260 |
| 43 | [Protein export](file:///H:\其他人转录组数据\魏永赞转录组\新数据\新数据\果皮转录组\附件\4-kegg-all%20genes.xlsx#RANGE!gene43) | 140 (0.79%) | ko03060 |
| 44 | [Phenylalanine, tyrosine and tryptophan biosynthesis](file:///H:\其他人转录组数据\魏永赞转录组\新数据\新数据\果皮转录组\附件\4-kegg-all%20genes.xlsx#RANGE!gene44) | 138 (0.78%) | ko00400 |
| 45 | [Fatty acid metabolism](file:///H:\其他人转录组数据\魏永赞转录组\新数据\新数据\果皮转录组\附件\4-kegg-all%20genes.xlsx#RANGE!gene45) | 135 (0.76%) | ko00071 |
| 46 | [Basal transcription factors](file:///H:\其他人转录组数据\魏永赞转录组\新数据\新数据\果皮转录组\附件\4-kegg-all%20genes.xlsx#RANGE!gene46) | 134 (0.76%) | ko03022 |
| 47 | [N-Glycan biosynthesis](file:///H:\其他人转录组数据\魏永赞转录组\新数据\新数据\果皮转录组\附件\4-kegg-all%20genes.xlsx#RANGE!gene47) | 133 (0.75%) | ko00510 |
| 48 | [Glycerolipid metabolism](file:///H:\其他人转录组数据\魏永赞转录组\新数据\新数据\果皮转录组\附件\4-kegg-all%20genes.xlsx#RANGE!gene48) | 131 (0.74%) | ko00561 |
| 49 | [alpha-Linolenic acid metabolism](file:///H:\其他人转录组数据\魏永赞转录组\新数据\新数据\果皮转录组\附件\4-kegg-all%20genes.xlsx#RANGE!gene49) | 129 (0.73%) | ko00592 |
| 50 | [Pentose and glucuronate interconversions](file:///H:\其他人转录组数据\魏永赞转录组\新数据\新数据\果皮转录组\附件\4-kegg-all%20genes.xlsx#RANGE!gene50) | 123 (0.7%) | ko00040 |
| 51 | [Nitrogen metabolism](file:///H:\其他人转录组数据\魏永赞转录组\新数据\新数据\果皮转录组\附件\4-kegg-all%20genes.xlsx#RANGE!gene51) | 122 (0.69%) | ko00910 |
| 52 | [beta-Alanine metabolism](file:///H:\其他人转录组数据\魏永赞转录组\新数据\新数据\果皮转录组\附件\4-kegg-all%20genes.xlsx#RANGE!gene52) | 120 (0.68%) | ko00410 |
| 53 | [Terpenoid backbone biosynthesis](file:///H:\其他人转录组数据\魏永赞转录组\新数据\新数据\果皮转录组\附件\4-kegg-all%20genes.xlsx#RANGE!gene53) | 117 (0.66%) | ko00900 |
| 54 | [Biosynthesis of unsaturated fatty acids](file:///H:\其他人转录组数据\魏永赞转录组\新数据\新数据\果皮转录组\附件\4-kegg-all%20genes.xlsx#RANGE!gene54) | 116 (0.66%) | ko01040 |
| 55 | [Porphyrin and chlorophyll metabolism](file:///H:\其他人转录组数据\魏永赞转录组\新数据\新数据\果皮转录组\附件\4-kegg-all%20genes.xlsx#RANGE!gene55) | 112 (0.63%) | ko00860 |
| 56 | [Propanoate metabolism](file:///H:\其他人转录组数据\魏永赞转录组\新数据\新数据\果皮转录组\附件\4-kegg-all%20genes.xlsx#RANGE!gene56) | 111 (0.63%) | ko00640 |
| 57 | [DNA replication](file:///H:\其他人转录组数据\魏永赞转录组\新数据\新数据\果皮转录组\附件\4-kegg-all%20genes.xlsx#RANGE!gene57) | 110 (0.62%) | ko03030 |
| 58 | [Tyrosine metabolism](file:///H:\其他人转录组数据\魏永赞转录组\新数据\新数据\果皮转录组\附件\4-kegg-all%20genes.xlsx#RANGE!gene58) | 108 (0.61%) | ko00350 |
| 59 | [Circadian rhythm - plant](file:///H:\其他人转录组数据\魏永赞转录组\新数据\新数据\果皮转录组\附件\4-kegg-all%20genes.xlsx#RANGE!gene59) | 105 (0.59%) | ko04712 |
| 60 | [Photosynthesis](file:///H:\其他人转录组数据\魏永赞转录组\新数据\新数据\果皮转录组\附件\4-kegg-all%20genes.xlsx#RANGE!gene60) | 101 (0.57%) | ko00195 |
| 61 | [RNA polymerase](file:///H:\其他人转录组数据\魏永赞转录组\新数据\新数据\果皮转录组\附件\4-kegg-all%20genes.xlsx#RANGE!gene61) | 98 (0.55%) | ko03020 |
| 62 | [Pantothenate and CoA biosynthesis](file:///H:\其他人转录组数据\魏永赞转录组\新数据\新数据\果皮转录组\附件\4-kegg-all%20genes.xlsx#RANGE!gene62) | 97 (0.55%) | ko00770 |
| 63 | [Ascorbate and aldarate metabolism](file:///H:\其他人转录组数据\魏永赞转录组\新数据\新数据\果皮转录组\附件\4-kegg-all%20genes.xlsx#RANGE!gene63) | 94 (0.53%) | ko00053 |
| 64 | [Valine, leucine and isoleucine biosynthesis](file:///H:\其他人转录组数据\魏永赞转录组\新数据\新数据\果皮转录组\附件\4-kegg-all%20genes.xlsx#RANGE!gene64) | 89 (0.5%) | ko00290 |
| 65 | [Base excision repair](file:///H:\其他人转录组数据\魏永赞转录组\新数据\新数据\果皮转录组\附件\4-kegg-all%20genes.xlsx#RANGE!gene65) | 87 (0.49%) | ko03410 |
| 66 | [SNARE interactions in vesicular transport](file:///H:\其他人转录组数据\魏永赞转录组\新数据\新数据\果皮转录组\附件\4-kegg-all%20genes.xlsx#RANGE!gene66) | 83 (0.47%) | ko04130 |
| 67 | [Tropane, piperidine and pyridine alkaloid biosynthesis](file:///H:\其他人转录组数据\魏永赞转录组\新数据\新数据\果皮转录组\附件\4-kegg-all%20genes.xlsx#RANGE!gene67) | 82 (0.46%) | ko00960 |
| 68 | [Butanoate metabolism](file:///H:\其他人转录组数据\魏永赞转录组\新数据\新数据\果皮转录组\附件\4-kegg-all%20genes.xlsx#RANGE!gene68) | 81 (0.46%) | ko00650 |
| 69 | [Cyanoamino acid metabolism](file:///H:\其他人转录组数据\魏永赞转录组\新数据\新数据\果皮转录组\附件\4-kegg-all%20genes.xlsx#RANGE!gene69) | 80 (0.45%) | ko00460 |
| 70 | [Homologous recombination](file:///H:\其他人转录组数据\魏永赞转录组\新数据\新数据\果皮转录组\附件\4-kegg-all%20genes.xlsx#RANGE!gene70) | 78 (0.44%) | ko03440 |
| 71 | [Fatty acid biosynthesis](file:///H:\其他人转录组数据\魏永赞转录组\新数据\新数据\果皮转录组\附件\4-kegg-all%20genes.xlsx#RANGE!gene71) | 78 (0.44%) | ko00061 |
| 72 | [Sphingolipid metabolism](file:///H:\其他人转录组数据\魏永赞转录组\新数据\新数据\果皮转录组\附件\4-kegg-all%20genes.xlsx#RANGE!gene72) | 76 (0.43%) | ko00600 |
| 73 | [One carbon pool by folate](file:///H:\其他人转录组数据\魏永赞转录组\新数据\新数据\果皮转录组\附件\4-kegg-all%20genes.xlsx#RANGE!gene73) | 76 (0.43%) | ko00670 |
| 74 | [Carotenoid biosynthesis](file:///H:\其他人转录组数据\魏永赞转录组\新数据\新数据\果皮转录组\附件\4-kegg-all%20genes.xlsx#RANGE!gene74) | 75 (0.42%) | ko00906 |
| 75 | [Regulation of autophagy](file:///H:\其他人转录组数据\魏永赞转录组\新数据\新数据\果皮转录组\附件\4-kegg-all%20genes.xlsx#RANGE!gene75) | 75 (0.42%) | ko04140 |
| 76 | [Ether lipid metabolism](file:///H:\其他人转录组数据\魏永赞转录组\新数据\新数据\果皮转录组\附件\4-kegg-all%20genes.xlsx#RANGE!gene76) | 74 (0.42%) | ko00565 |
| 77 | [Tryptophan metabolism](file:///H:\其他人转录组数据\魏永赞转录组\新数据\新数据\果皮转录组\附件\4-kegg-all%20genes.xlsx#RANGE!gene77) | 74 (0.42%) | ko00380 |
| 78 | [Mismatch repair](file:///H:\其他人转录组数据\魏永赞转录组\新数据\新数据\果皮转录组\附件\4-kegg-all%20genes.xlsx#RANGE!gene78) | 70 (0.4%) | ko03430 |
| 79 | [Glycosylphosphatidylinositol(GPI)-anchor biosynthesis](file:///H:\其他人转录组数据\魏永赞转录组\新数据\新数据\果皮转录组\附件\4-kegg-all%20genes.xlsx#RANGE!gene79) | 69 (0.39%) | ko00563 |
| 80 | [Sulfur metabolism](file:///H:\其他人转录组数据\魏永赞转录组\新数据\新数据\果皮转录组\附件\4-kegg-all%20genes.xlsx#RANGE!gene80) | 68 (0.38%) | ko00920 |
| 81 | [Ubiquinone and other terpenoid-quinone biosynthesis](file:///H:\其他人转录组数据\魏永赞转录组\新数据\新数据\果皮转录组\附件\4-kegg-all%20genes.xlsx#RANGE!gene81) | 64 (0.36%) | ko00130 |
| 82 | [Natural killer cell mediated cytotoxicity](file:///H:\其他人转录组数据\魏永赞转录组\新数据\新数据\果皮转录组\附件\4-kegg-all%20genes.xlsx#RANGE!gene82) | 61 (0.34%) | ko04650 |
| 83 | [Histidine metabolism](file:///H:\其他人转录组数据\魏永赞转录组\新数据\新数据\果皮转录组\附件\4-kegg-all%20genes.xlsx#RANGE!gene83) | 61 (0.34%) | ko00340 |
| 84 | [Isoquinoline alkaloid biosynthesis](file:///H:\其他人转录组数据\魏永赞转录组\新数据\新数据\果皮转录组\附件\4-kegg-all%20genes.xlsx#RANGE!gene84) | 60 (0.34%) | ko00950 |
| 85 | [Steroid biosynthesis](file:///H:\其他人转录组数据\魏永赞转录组\新数据\新数据\果皮转录组\附件\4-kegg-all%20genes.xlsx#RANGE!gene85) | 60 (0.34%) | ko00100 |
| 86 | [Lysine degradation](file:///H:\其他人转录组数据\魏永赞转录组\新数据\新数据\果皮转录组\附件\4-kegg-all%20genes.xlsx#RANGE!gene86) | 58 (0.33%) | ko00310 |
| 87 | [Selenocompound metabolism](file:///H:\其他人转录组数据\魏永赞转录组\新数据\新数据\果皮转录组\附件\4-kegg-all%20genes.xlsx#RANGE!gene87) | 57 (0.32%) | ko00450 |
| 88 | [Other glycan degradation](file:///H:\其他人转录组数据\魏永赞转录组\新数据\新数据\果皮转录组\附件\4-kegg-all%20genes.xlsx#RANGE!gene88) | 52 (0.29%) | ko00511 |
| 89 | [Linoleic acid metabolism](file:///H:\其他人转录组数据\魏永赞转录组\新数据\新数据\果皮转录组\附件\4-kegg-all%20genes.xlsx#RANGE!gene89) | 51 (0.29%) | ko00591 |
| 90 | [Folate biosynthesis](file:///H:\其他人转录组数据\魏永赞转录组\新数据\新数据\果皮转录组\附件\4-kegg-all%20genes.xlsx#RANGE!gene90) | 49 (0.28%) | ko00790 |
| 91 | [Fatty acid elongation in mitochondria](file:///H:\其他人转录组数据\魏永赞转录组\新数据\新数据\果皮转录组\附件\4-kegg-all%20genes.xlsx#RANGE!gene91) | 44 (0.25%) | ko00062 |
| 92 | [Nicotinate and nicotinamide metabolism](file:///H:\其他人转录组数据\魏永赞转录组\新数据\新数据\果皮转录组\附件\4-kegg-all%20genes.xlsx#RANGE!gene92) | 44 (0.25%) | ko00760 |
| 93 | [Limonene and pinene degradation](file:///H:\其他人转录组数据\魏永赞转录组\新数据\新数据\果皮转录组\附件\4-kegg-all%20genes.xlsx#RANGE!gene93) | 42 (0.24%) | ko00903 |
| 94 | [Photosynthesis - antenna proteins](file:///H:\其他人转录组数据\魏永赞转录组\新数据\新数据\果皮转录组\附件\4-kegg-all%20genes.xlsx#RANGE!gene94) | 41 (0.23%) | ko00196 |
| 95 | [Taurine and hypotaurine metabolism](file:///H:\其他人转录组数据\魏永赞转录组\新数据\新数据\果皮转录组\附件\4-kegg-all%20genes.xlsx#RANGE!gene95) | 39 (0.22%) | ko00430 |
| 96 | [Lysine biosynthesis](file:///H:\其他人转录组数据\魏永赞转录组\新数据\新数据\果皮转录组\附件\4-kegg-all%20genes.xlsx#RANGE!gene96) | 39 (0.22%) | ko00300 |
| 97 | [Arachidonic acid metabolism](file:///H:\其他人转录组数据\魏永赞转录组\新数据\新数据\果皮转录组\附件\4-kegg-all%20genes.xlsx#RANGE!gene97) | 35 (0.2%) | ko00590 |
| 98 | [Flavonoid biosynthesis](file:///H:\其他人转录组数据\魏永赞转录组\新数据\新数据\果皮转录组\附件\4-kegg-all%20genes.xlsx#RANGE!gene98) | 34 (0.19%) | ko00941 |
| 99 | [Circadian rhythm - mammal](file:///H:\其他人转录组数据\魏永赞转录组\新数据\新数据\果皮转录组\附件\4-kegg-all%20genes.xlsx#RANGE!gene99) | 34 (0.19%) | ko04710 |
| 100 | [Sulfur relay system](file:///H:\其他人转录组数据\魏永赞转录组\新数据\新数据\果皮转录组\附件\4-kegg-all%20genes.xlsx#RANGE!gene100) | 34 (0.19%) | ko04122 |
| 101 | [Riboflavin metabolism](file:///H:\其他人转录组数据\魏永赞转录组\新数据\新数据\果皮转录组\附件\4-kegg-all%20genes.xlsx#RANGE!gene101) | 33 (0.19%) | ko00740 |
| 102 | [Non-homologous end-joining](file:///H:\其他人转录组数据\魏永赞转录组\新数据\新数据\果皮转录组\附件\4-kegg-all%20genes.xlsx#RANGE!gene102) | 30 (0.17%) | ko03450 |
| 103 | [Thiamine metabolism](file:///H:\其他人转录组数据\魏永赞转录组\新数据\新数据\果皮转录组\附件\4-kegg-all%20genes.xlsx#RANGE!gene103) | 29 (0.16%) | ko00730 |
| 104 | [Glycosaminoglycan degradation](file:///H:\其他人转录组数据\魏永赞转录组\新数据\新数据\果皮转录组\附件\4-kegg-all%20genes.xlsx#RANGE!gene104) | 27 (0.15%) | ko00531 |
| 105 | [Other types of O-glycan biosynthesis](file:///H:\其他人转录组数据\魏永赞转录组\新数据\新数据\果皮转录组\附件\4-kegg-all%20genes.xlsx#RANGE!gene105) | 26 (0.15%) | ko00514 |
| 106 | [Vitamin B6 metabolism](file:///H:\其他人转录组数据\魏永赞转录组\新数据\新数据\果皮转录组\附件\4-kegg-all%20genes.xlsx#RANGE!gene106) | 26 (0.15%) | ko00750 |
| 107 | [Glycosphingolipid biosynthesis - globo series](file:///H:\其他人转录组数据\魏永赞转录组\新数据\新数据\果皮转录组\附件\4-kegg-all%20genes.xlsx#RANGE!gene107) | 26 (0.15%) | ko00603 |
| 108 | [Glucosinolate biosynthesis](file:///H:\其他人转录组数据\魏永赞转录组\新数据\新数据\果皮转录组\附件\4-kegg-all%20genes.xlsx#RANGE!gene108) | 25 (0.14%) | ko00966 |
| 109 | [Synthesis and degradation of ketone bodies](file:///H:\其他人转录组数据\魏永赞转录组\新数据\新数据\果皮转录组\附件\4-kegg-all%20genes.xlsx#RANGE!gene109) | 24 (0.14%) | ko00072 |
| 110 | [Zeatin biosynthesis](file:///H:\其他人转录组数据\魏永赞转录组\新数据\新数据\果皮转录组\附件\4-kegg-all%20genes.xlsx#RANGE!gene110) | 20 (0.11%) | ko00908 |
| 111 | [Monoterpenoid biosynthesis](file:///H:\其他人转录组数据\魏永赞转录组\新数据\新数据\果皮转录组\附件\4-kegg-all%20genes.xlsx#RANGE!gene111) | 18 (0.1%) | ko00902 |
| 112 | [Flavone and flavonol biosynthesis](file:///H:\其他人转录组数据\魏永赞转录组\新数据\新数据\果皮转录组\附件\4-kegg-all%20genes.xlsx#RANGE!gene112) | 18 (0.1%) | ko00944 |
| 113 | [Stilbenoid, diarylheptanoid and gingerol biosynthesis](file:///H:\其他人转录组数据\魏永赞转录组\新数据\新数据\果皮转录组\附件\4-kegg-all%20genes.xlsx#RANGE!gene113) | 18 (0.1%) | ko00945 |
| 114 | [Glycosphingolipid biosynthesis - ganglio series](file:///H:\其他人转录组数据\魏永赞转录组\新数据\新数据\果皮转录组\附件\4-kegg-all%20genes.xlsx#RANGE!gene114) | 14 (0.08%) | ko00604 |
| 115 | [Caffeine metabolism](file:///H:\其他人转录组数据\魏永赞转录组\新数据\新数据\果皮转录组\附件\4-kegg-all%20genes.xlsx#RANGE!gene115) | 13 (0.07%) | ko00232 |
| 116 | [C5-Branched dibasic acid metabolism](file:///H:\其他人转录组数据\魏永赞转录组\新数据\新数据\果皮转录组\附件\4-kegg-all%20genes.xlsx#RANGE!gene116) | 12 (0.07%) | ko00660 |
| 117 | [Diterpenoid biosynthesis](file:///H:\其他人转录组数据\魏永赞转录组\新数据\新数据\果皮转录组\附件\4-kegg-all%20genes.xlsx#RANGE!gene117) | 11 (0.06%) | ko00904 |
| 118 | [Benzoxazinoid biosynthesis](file:///H:\其他人转录组数据\魏永赞转录组\新数据\新数据\果皮转录组\附件\4-kegg-all%20genes.xlsx#RANGE!gene118) | 10 (0.06%) | ko00402 |
| 119 | [Sesquiterpenoid biosynthesis](file:///H:\其他人转录组数据\魏永赞转录组\新数据\新数据\果皮转录组\附件\4-kegg-all%20genes.xlsx#RANGE!gene119) | 9 (0.05%) | ko00909 |
| 120 | [Biotin metabolism](file:///H:\其他人转录组数据\魏永赞转录组\新数据\新数据\果皮转录组\附件\4-kegg-all%20genes.xlsx#RANGE!gene120) | 8 (0.05%) | ko00780 |
| 121 | [ABC transporters](file:///H:\其他人转录组数据\魏永赞转录组\新数据\新数据\果皮转录组\附件\4-kegg-all%20genes.xlsx#RANGE!gene121) | 7 (0.04%) | ko02010 |
| 122 | [Brassinosteroid biosynthesis](file:///H:\其他人转录组数据\魏永赞转录组\新数据\新数据\果皮转录组\附件\4-kegg-all%20genes.xlsx#RANGE!gene122) | 7 (0.04%) | ko00905 |
| 123 | [Lipoic acid metabolism](file:///H:\其他人转录组数据\魏永赞转录组\新数据\新数据\果皮转录组\附件\4-kegg-all%20genes.xlsx#RANGE!gene123) | 6 (0.03%) | ko00785 |
| 124 | [Anthocyanin biosynthesis](file:///H:\其他人转录组数据\魏永赞转录组\新数据\新数据\果皮转录组\附件\4-kegg-all%20genes.xlsx#RANGE!gene124) | 4 (0.02%) | ko00942 |
| 125 | [Betalain biosynthesis](file:///H:\其他人转录组数据\魏永赞转录组\新数据\新数据\果皮转录组\附件\4-kegg-all%20genes.xlsx#RANGE!gene125) | 1 (0.01%) | ko00965 |


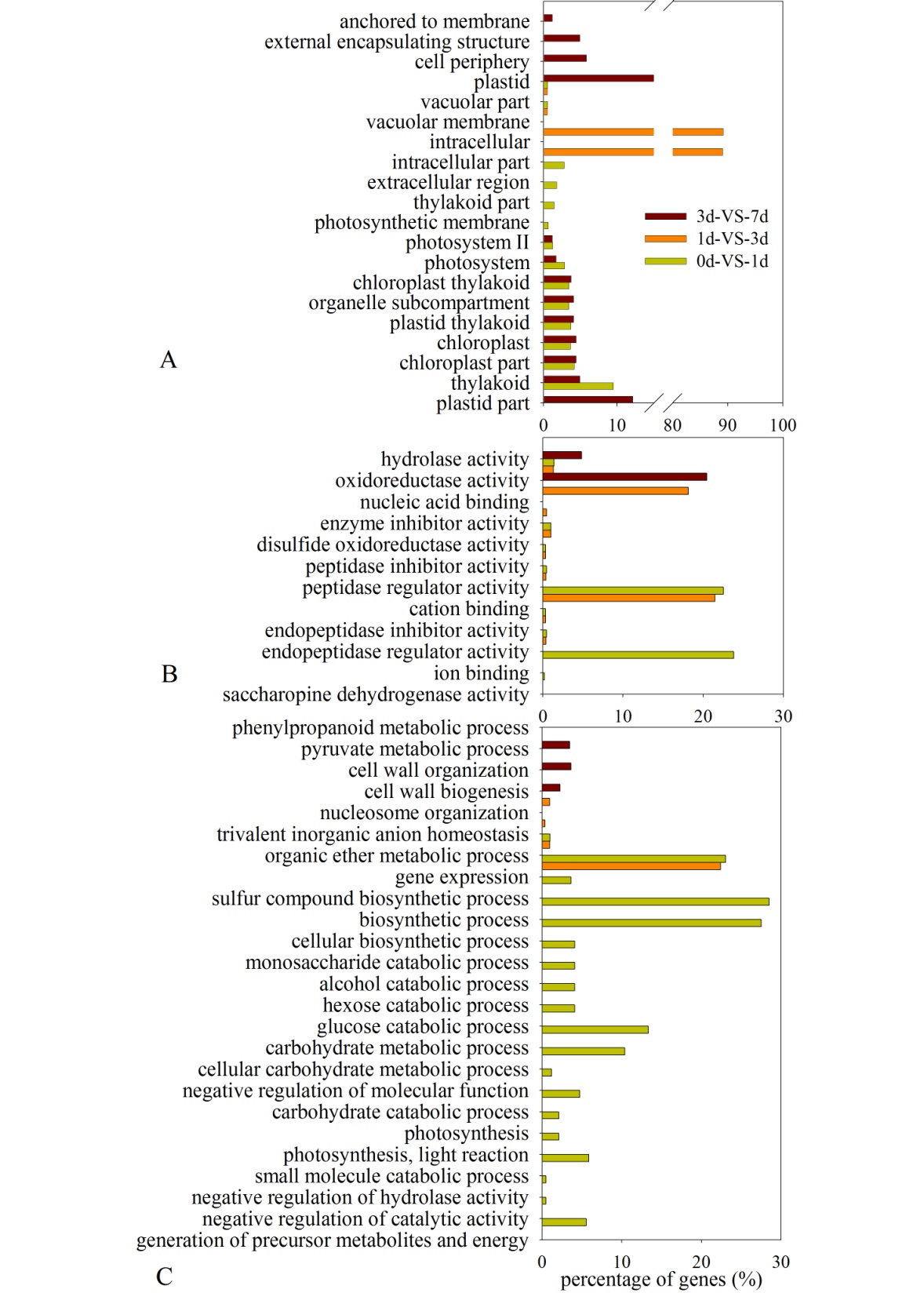


**Additional file 5.** Functional categories of DGEs based on Gene Ontology (GO). Significantly enriched GO categories (P-value < 0.05) were analyzed in pairwise comparisons (0d-VS-1d, 1d-VS-3d, and 3d-VS-7d). The results are summarized in three main categories: (A) cellular component, (B) molecular function, and (C) biological process. The y-axis indicates the percentage of a specific category of unigenes, which are based on the proportion of the genes in each set.

**Additional File 6.** Pathway assignment based on KEGG by 0d-VS-1d, 1d-VS-3d, and 3d-VS-7d comparisons.

| **0dVS1d** |  | **KEGG pathways significantly enriched** | **Number of DEGs** | **P-value** | **Q-value** | **Pathway ID** |
| --- | --- | --- | --- | --- | --- | --- |
|  | 1 | Photosynthesis | 41 | 1.75E-12 | 2.07E-10 | ko00195 |
|  | 2 | Photosynthesis - antenna proteins | 18 | 7.50E-07 | 4.42E-05 | ko00196 |
|  | 3 | Biosynthesis of secondary metabolites | 350 | 6.78E-06 | 2.67E-04 | ko01110 |
|  | 4 | Flavonoid biosynthesis | 13 | 1.48E-04 | 4.35E-03 | ko00941 |
|  | 5 | Peroxisome | 48 | 3.13E-04 | 7.38E-03 | ko04146 |
|  | 6 | Metabolic pathways | 645 | 3.79E-04 | 7.46E-03 | ko01100 |
|  | 7 | Glyoxylate and dicarboxylate metabolism | 42 | 6.28E-04 | 1.06E-02 | ko00630 |
|  | 8 | Phenylpropanoid biosynthesis | 33 | 2.00E-03 | 2.95E-02 | ko00940 |
|  | 9 | Caffeine metabolism | 6 | 3.20E-03 | 4.20E-02 | ko00232 |
|  | All DEGs with pathway annotation | | 1196 |  |  |  |
| **1dVS3d** | 1 | Protein processing in endoplasmic reticulum | 141 | 1.83E-08 | 2.22E-06 | ko04141 |
|  | 2 | Peroxisome | 56 | 3.32E-04 | 2.01E-02 | ko04146 |
|  | 3 | Fatty acid metabolism | 36 | 6.33E-04 | 2.55E-02 | ko00071 |
|  | 4 | Circadian rhythm - plant | 29 | 1.13E-03 | 3.41E-02 | ko04712 |
|  | 5 | Caffeine metabolism | 7 | 1.59E-03 | 3.85E-02 | ko00232 |
|  | 6 | Glyoxylate and dicarboxylate metabolism | 47 | 2.09E-03 | 4.08E-02 | ko00630 |
|  | 7 | Lysine degradation | 18 | 2.36E-03 | 4.08E-02 | ko00310 |
|  | All DEGs with pathway annotation | | 334 |  |  |  |
| **3dVS7d** | 1 | Phenylpropanoid biosynthesis | 29 | 5.96E-12 | 6.26E-10 | ko00940 |
|  | 2 | Photosynthesis | 19 | 2.19E-08 | 1.15E-06 | ko00195 |
|  | 3 | Phenylalanine metabolism | 23 | 6.17E-08 | 2.16E-06 | ko00360 |
|  | 4 | Metabolic pathways | 243 | 8.39E-07 | 2.20E-05 | ko01100 |
|  | 5 | Biosynthesis of secondary metabolites | 130 | 1.47E-05 | 3.08E-04 | ko01110 |
|  | 6 | Flavonoid biosynthesis | 8 | 5.18E-05 | 9.07E-04 | ko00941 |
|  | 7 | Zeatin biosynthesis | 6 | 1.07E-04 | 1.49E-03 | ko00908 |
|  | 8 | Starch and sucrose metabolism | 31 | 1.14E-04 | 1.49E-03 | ko00500 |
|  | 9 | Pentose and glucuronate interconversions | 15 | 1.45E-04 | 1.62E-03 | ko00040 |
|  | 10 | Glutathione metabolism | 19 | 1.54E-04 | 1.62E-03 | ko00480 |
|  | 11 | Protein processing in endoplasmic reticulum | 41 | 4.52E-04 | 4.32E-03 | ko04141 |
|  | 12 | Photosynthesis - antenna proteins | 7 | 1.22E-03 | 1.06E-02 | ko00196 |
|  | 13 | Cyanoamino acid metabolism | 10 | 1.49E-03 | 1.20E-02 | ko00460 |
|  | 14 | Circadian rhythm - plant | 11 | 3.67E-03 | 2.75E-02 | ko04712 |
|  | 15 | Plant-pathogen interaction | 33 | 5.66E-03 | 3.96E-02 | ko04626 |
|  | All DEGs with pathway annotation | | 625 |  |  |  |

**
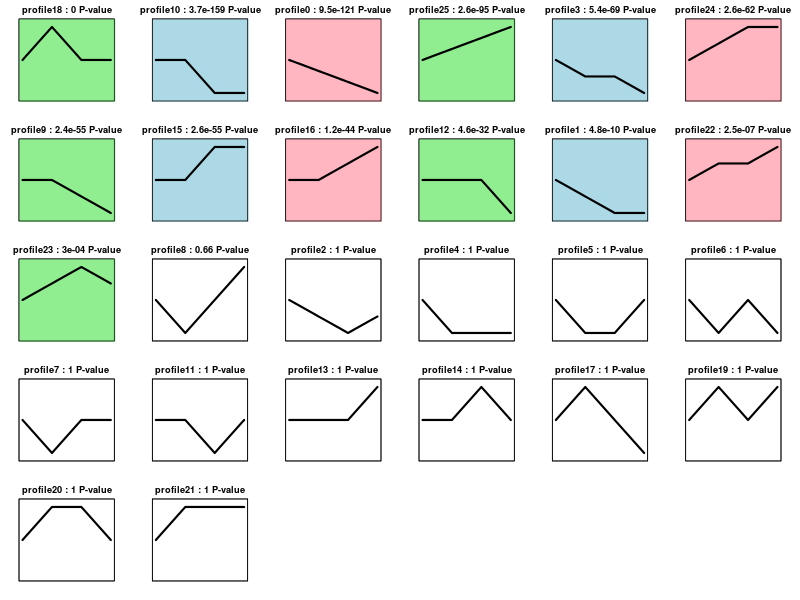
**

**Additional File 7.** Clusters ordered based on number of genes and profiles by significance. The transcripts were divided into 25 clusters representing distinct expression patterns, and 13 clusters with significant differential expression at P-value < 0.05.
